# Small molecule angiotensin converting enzyme inhibitors: A medicinal chemistry perspective

**DOI:** 10.3389/fphar.2022.968104

**Published:** 2022-11-01

**Authors:** Wenyue Zheng, Erkang Tian, Zhen Liu, Changhan Zhou, Pei Yang, Keyue Tian, Wen Liao, Juan Li, Changyu Ren

**Affiliations:** ^1^ Departments of Obstetrics & Gynecology and Pediatrics, Key Laboratory of Birth Defects and Related Diseases of Women and Children, Ministry of Education, West China Second University Hospital, Sichuan University, Chengdu, China; ^2^ Health Management Center, West China Second University Hospital, Chengdu, China; ^3^ State Key Laboratory of Oral Diseases, National Clinical Research Center for Oral Diseases & Department of Orthodontics, West China Hospital of Stomatology, Sichuan University, Chengdu, China; ^4^ Department of Pharmacy, Chengdu Fifth People’s Hospital, Chengdu, China

**Keywords:** ACE—angiotensin-converting enzyme, inhibitors, drug design, structure-activity relationships (SAR), crystal structure

## Abstract

Angiotensin-converting enzyme (ACE), a zinc metalloprotein, is a central component of the renin–angiotensin system (RAS). It degrades bradykinin and other vasoactive peptides. Angiotensin-converting-enzyme inhibitors (ACE inhibitors, ACEIs) decrease the formation of angiotensin II and increase the level of bradykinin, thus relaxing blood vessels as well as reducing blood volume, lowering blood pressure and reducing oxygen consumption by the heart, which can be used to prevent and treat cardiovascular diseases and kidney diseases. Nevertheless, ACEIs are associated with a range of adverse effects such as renal insufficiency, which limits their use. In recent years, researchers have attempted to reduce the adverse effects of ACEIs by improving the selectivity of ACEIs for structural domains based on conformational relationships, and have developed a series of novel ACEIs. In this review, we have summarized the research advances of ACE inhibitors, focusing on the development sources, design strategies and analysis of structure-activity relationships and the biological activities of ACE inhibitors.

## Graphical Abstract

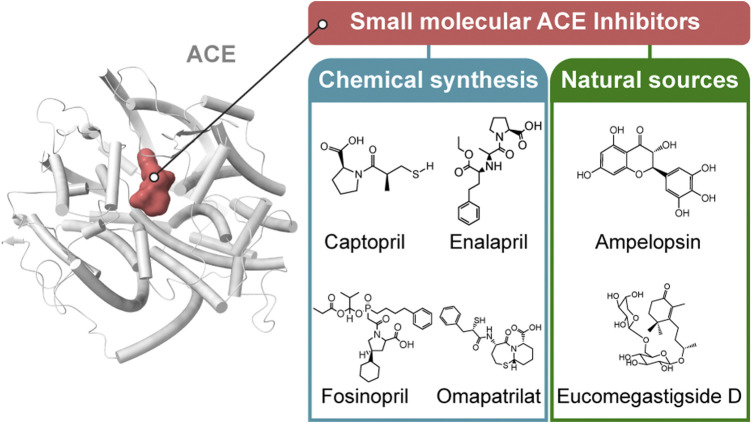

## 1 Introduction

The main function of ACE is to convert the hormone angiotensin I into active angiotensin II (Turner and Hooper, 2002; Fukuda and Sata, 2008) and to degrade bradykinin (a vasodilator), which constricts blood vessels and leads to an increase in blood pressure. In addition, the classical RAS driven by ACE has been shown to be associated with a variety of diseases such as chronic heart disease, kidney disease and diabetes (Warner et al., 2020).

Angiotensin-converting-enzyme inhibitors (ACE inhibitors, ACEIs) are widely used to lower blood pressure and reduce cardiac oxygen consumption (Herman et al., 2021). Based on their inhibition of renin-angiotensin-aldosterone system (RAAS) activity, ACEIs can help treat hypertension (James et al., 2014), acute myocardial infarction (heart disease) (O’Gara et al., 2013), heart failure (Ponikowski et al., 2016; Yancy et al., 2017), renal complications of diabetes mellitus (diabetic nephropathy) (Strippoli et al., 2005; Zhang Y. et al., 2020), etc. They have also been used to help decrease psychogenic polydipsia and to manage posttransplant erythrocytosis. In addition, ACEIs may also be used as adjuncts to the treatment of patients with malignancies (Rosenthal and Gavras, 2019). Notably, ACEIs are often used in combination with other drugs to improve outcomes, such as with angiotensin receptor blocker (ARB) (known as dual blockade) to improve existing therapies, and with calcium channel blocker (CCB) for arterial hypertension (Redón et al., 2013).

However some adverse effects remain with current ACEIs formulations, common ones include first dose hypotension, renal dysfunction, hyperkalaemia and cough (Alderman, 1996). Less common side effects include angioedema (Kostis et al., 2018) and liver toxicity (Douros et al., 2013). There are also studies that suggest that the use of ACEiACEIs can lead to gingival overgrowth (Ustaoğlu et al., 2021) and angioedema of the tongue (Duvančić et al., 2011). ACEIs have also been associated with adverse fetal reactions (Tabacova, 2005) (Bullo et al., 2012). In recent years, researchers have attempted to reduce the adverse effects of ACEIs by improving their selectivity for structural domains based on conformational relationships, and a series of novel ACEIs have been developed.

Focusing on the development sources, design strategies and analysis of structure-activity relationships and the biological activities of ACE inhibitors, we have summarized the research advances of ACE inhibitors. We hope that this Perspective can provide a summary of the status in the field of ACE inhibitors along with potential pathways for further advances.

## 2 Domain structures and biological functions of ACE and ACE inhibitors

### 2.1 Domain structures of ACE

Angiotensin converting enzyme (ACE) is a chlorine-and zinc-dependent polypeptide dipeptidase (Corradi et al., 2006). Human angiotensin converting enzyme consists of two isoforms: the longer somatic angiotensin converting enzyme (sACE) and the shorter testicular angiotensin converting enzyme (tACE) (Corradi et al., 2006). sACE has two catalytic domains (ACE-N and ACE-C) and tACE has one catalytic domain (ACE-C). The N and C domains of sACE share 60% sequence identity and therefore share the same overall topology as well as a highly conserved zinc-binding motif (HEXXH) closely related to catalytic activity (Corradi et al., 2006). The researchers compared the differences between the two catalytic domains, ACE-C and ACE-N, by superimposing sACE and tACE (Corradi et al., 2006). When superimposed, the most noticeable difference between the N and C domains is the extra length of the ACE-N at the N and C terminus, which includes the interdomain linker (Corradi et al., 2006).

### 2.2 Biological function of ACE

**FIGURE 1:**
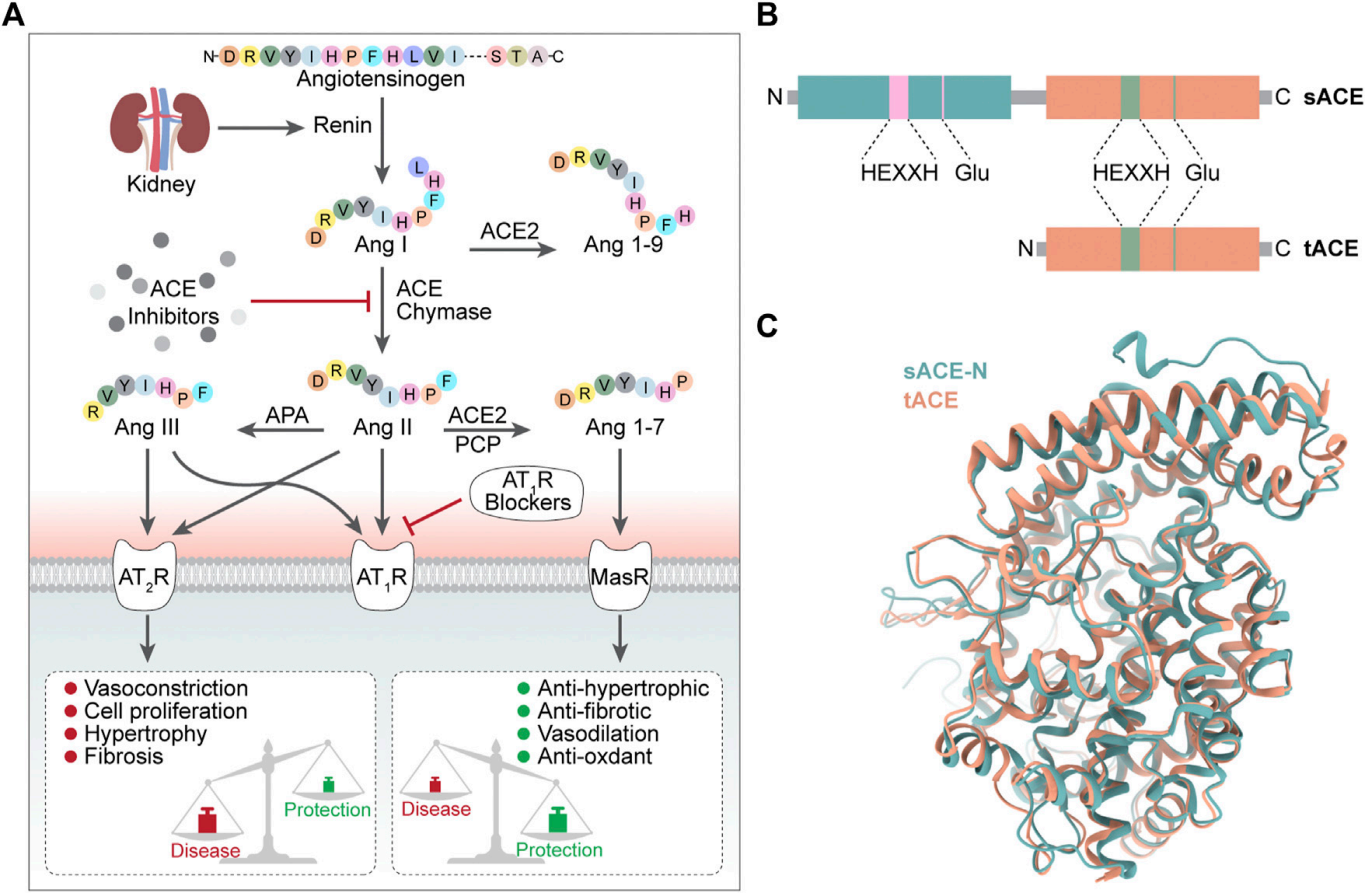
**(A)** Conversion process and biology function of ACE **(B)** Domain structure of sACE and tACE **(C)** 3D structure of sACE sACE-N is in green. sACE-C (tACE) is in orange.

ACE hydrolyses the Phe8-His9 peptide bond of decapeptide AngI to release a C-terminal dipeptide His-Leu and the octapeptide AngII, a vasoconstrictor, in the presence of zinc ions (Zhao and Xu, 2008; Khurana and Goswami, 2022). ACE also degrades bradykinin (a potent vasodilator). In addition, ACE catalyzes the conversion of angiotensin (1–9) to angiotensin (1–7) (Campbell et al., 2004) and plays a role in the endogenous counterregulatory pathway within the RAS (Santos et al., 2013) (Figure 1).

Notably, the classical RAS driven by ACE has been shown to be associated with tissue fibrosis in many diseases, including chronic heart disease, kidney disease and diabetes (Warner et al., 2020), and the protective RAS regulated by ACE2 could counteract or modulate these effects and is a promising direction for antifibrotic therapy (Mak et al., 2015; Santos et al., 2018).

The physiological function of ACE is achieved mainly through two catalytic domains (ACE-N and ACE-C) (Lin et al., 2022), which do not have identical roles. The ACE C-domain plays a role in converting Ang I to Ang II (Iwaniak et al., 2014). Studies have shown that in inactivated mice with N domains, the C domain alone can produce enough Ang II *in vivo* to be as effective as wild-type enzymes (or almost as effective), while mice lacking C domain activity respond poorly to dehydration stress and cannot concentrate urine as efficiently as mice of wide type (van Esch et al., 2005; Fuchs et al., 2008). TIn terms of bradykinin metabolism, the situation was not the same, as there was no significant difference in bradykinin levels between wild-type and N or C structural domain inactivated mice (Fuchs et al., 2008), probably due to the presence of various bradykinin-degrading enzymes, such as aminopeptidase, carboxypeptidase and neutral endopeptidase, in addition to ACE (Skidgel, 1992).

### 2.3 Biological functions of ACEI

ACEIs are commonly used in the clinical cardiovascular system and are also used to treat kidney disease. Angiotensin II is a powerful vasoconstrictor and stimulator of aldosterone release, and ACEIs accelerate its creation from angiotensin I (Neves et al., 2018).

#### 2.3.1 Treatment of hypertension

ACEIs are widely used to treat essential hypertension, the main mechanism of which is to control hypertension by inhibiting the biosynthesis of angiotensin II. Several ACEI drugs have been developed, such as ramipril (trade name: Altace), captopril (Capoten), enalapril (Vasotec)**.** A study has demonstrated that all of these drugs have significant hypotensive effects (Heran et al., 2008). Currently, ACEIs are often combinedly used with ARBs (Zhang Y. et al., 2020; Sinnott et al., 2020) or CCBs (Egan, 2007) to improve outcomes. Hypertension and atrial fibrillation (AF) often coexist in the same individual (Verdecchia et al., 2018). What’s more, ACEI may reduce new onset AF in hypertensive patients (Hsieh et al., 2016).

#### 2.3.2 Treatment of heart disease

ACEIs have been used as adjunctive therapy in persistent AF. Azfar G Zaman et alconcluded that long-term ACEI medication enabled electrical defibrillation and helped sustain sinus rhythm in patients with persistent AF (Kumagai, 2007). In addition, preablation use of an ACEI in patients who had nonparoxysmal AF with low LVEF has been found to improve ablation outcomes (Mohanty et al., 2015). ACEI can be used not only as an adjunct to the treatment of persistent AF but also to inhibit the progression of paroxysmal to persistent AF (Hirayama et al., 2005).

ACEIs can reduce and treat myocardial infarction (Evans et al., 2016; Nordenskjöld et al., 2021). Myocardial fibrin are more likely to be degraded by matrix metalloproteinase (MMPs) and its tissue inhibitors on patients with acute myocardial infarction (MI) (Papadopoulos et al., 2005). ACEIs inhibit MMPs and thereby inhibit the expansion of myocardial infarction foci (Papadopoulos et al., 2004). ACEIs also provide protection to the transplanted heart by modulating the vascular tone and endothelial function of the original coronary arteries (Steinhauff et al., 2004). In the circulating blood of patients with stable angina, ramipril can increase the number and activity of endothelial progenitor cells which are important cells involved in the repair of damage after cardiac ischemia (Fearon et al., 2017; Arashi et al., 2020).

#### 2.3.3 Treatment of kidney disease

ACE inhibitors have been proven to have some nephroprotective properties. An investigation found that long-term usage of ACEIs or ARBs (at least 12 mon) provided extra benefits in terms of retaining residual kidney function in CAPD (continuous ambulatory peritoneal dialysis) patients when compared to other antihypertensive medicines (Perini et al., 2020). The appearance of proteinuria and glomerular sclerosis after nephrectomy is often due to compensatory increased glomerular volume (GV) (Fukuda et al., 2012; D’Agati, 2017). ACEIs slow or prevent glomerular enlargement after nephrectomy by reducing renal hyperfiltration while selectively reducing excess IGF-1 (Naik et al., 2021). The most serious long-term complication of hemolytic-uremic syndrome (HUS) (Caletti et al., 2004) is renal damage. One study showed that patients with hemolytic uremia with early protein restriction and ACEI use had a better long-term prognosis (Caletti et al., 2004).

#### 2.3.4 Others

The antitumour effects of ACEIs have gained attention in recent years, such as in colorectal (Engineer et al., 2013; Ozawa et al., 2019), smooth muscle (Fischer et al., 2021), breast and pancreatic cancers (Chauhan et al., 2013). The main antitumour mechanisms of ACEIs include reduction of cancer-associated fibroblasts (CAFs) and extracellular matrix (ECM), regulation of immune cells and improvement of hypoxia (Wzgarda et al., 2017; Yang et al., 2021).

ACEIs can also be used to treat other conditions, such as posttransplant erythrocytosis (PTE). Clinical studies have demonstrated that low doses of ramipril normalize the hematocrit of most patients with PTE(R et al., 2007). In neurological disorders, ACEIs can reduce the rate of cognitive decline in patients with Alzheimer’s disease, prevent migraines, and treat patients with Parkinson’s disease by affecting dopamine levels (Wzgarda et al., 2017).

#### 2.3.5 Treatment in patients with COVID-19

During the COVID-19 pandemic, it was hypothesized that patients with COVID-19 treated with ACEIs might have a poorer prognosis, but analysis by several authors has concluded that there is currently no strong evidence that ACEI/ARB exposure is harmful in patients with COVID-19 infection (Kai and Kai, 2020, 19; Rico-Mesa et al., 2020; Sriram and Insel, 2020; Yang G. et al., 2020; Zhang X. et al., 2020). Furthermore, the usage of ACEI medications has been shown to improve the prognosis and reduce complications caused by COVID-19 (Oz et al., 2021). Hypertension slows viral clearance and exacerbates excessive airway inflammation in patients with COVID-19, while ACEI treatment has been associated with reducing excessive inflammation associated with COVID-19 and enhanced intracellular antiviral response (Trump et al., 2021). A multicenter study revealed the role of ACEIs/ARB in reducing the risk of all-cause death in hospitalized patients and patients with hypertension associated with COVID-19 (Zhang P. et al., 2020). Another study found that inpatient usage of ACEIs/ARBs was related with a lower risk of intestinal involvement and mortality among hospitalized patients with COVID-19 and coexisting hypertension as compared to individuals who were not on ACEIs/ARBs(Tan et al., 2020; Singh et al., 2021; Biswas et al., 2022). In conclusion, we do not recommend stopping ACEIs in patients with COVID-19.

### 2.4 Limitations of ACEI

Important adverse effects of ACEIs include hypotension with a single dose, renal insufficiency, hyperkalemia, and cough (Alderman, 1996). ACE inhibitors reduce systemic vascular resistance among people with chronic kidney disease while also leading to decreased filtration pressure and adverse renal effects, such as acute renal failure. Reduced aldosterone concentrations, reduced salt delivery to the distal kidney, altered collecting tubule function, poor excretion of potassium and high levels of potassium intake are the main mechanisms by which ACEs produce hyperkalemia (Navis et al., 1996; Weir and Rolfe, 2010). The protussive mediator bradykinin and substance P are considered to be important links in ACEI inducing cough (Dicpinigaitis, 2006). ACE can degrade bradykinin and substance P, and ACEI may allow the accumulation of these two substances in the airways, leading to sensitization of airway sensory nerves, resulting in contraction of airway smooth muscle and triggering bronchoconstriction and cough (Pinto et al., 2020). In addition, less common side effects of ACEIs include angioedema (Kostis et al., 2018) and hepatotoxicity (Douros et al., 2013). ACEIs are also associated with adverse pregnancy outcomes, such as renal complications, neurodevelopmental delays and developmental delays in the fetus (Tabacova, 2005; Bullo et al., 2012).

## 3 ACE inhibitors

Most of the currently available ACE inhibitors are short peptides and their derivatives. The core of ACE inhibitors is a group chelating with the divalent zinc ion. In lisinopril and enalaprilat, this group is a carboxyl. In fosinopril, this group is a phosphonate. The residues of ACE (P1, P1′, P2, P2′, Pn) bind with the subsite of ACE (S1, S2, S1′, S2′) through various interactions, such as hydrogen bonds, and differences in the residues lead to different affinities for the ACE domain. The ACE inhibitors and their surrounding residues are listed in the Table1.

**TABLE 1:** ACE inhibitors and residues.

Inhibitors	Surrounding residues in ACE-C	Surrounding residues in ACE-N
Captopril	Q281, H353, A354, V380, H383, E384, H387, E411, F457, K511, H513, Y520, Y523, F527	Q259, H331, A332, T358, H361, E362, H365, E389, F435, K489, H491, Y498, Y501, F505
Lisinopril	E162, Q281, H353, A354, S355, D377, V380, H383, E384, H387, E411, F457, K511, F512, H513, S516, V518, Y520, Y523, F527	D140, Q259, H331, A332, S333, Q355, T358, H361, E362, H365, E389, F435, K489, F490, H491, N494, T496,Y498, Y501, F505
Enalapril	Q281, H353, A354, S355, V380, H383, E384, H387, E411, F457, K511, F512, H513, S516, V518, Y520,Y523	Q259, H331, A332, S333, T358, H361, E362, H365, E389, F435, K489, F490, H491, N494, T496, Y498, Y501, F505
Ramiprilat	Q281, T282, H353, A354, S355, V380, H383, E384, H387, E411, D453, F457, K511, F512, H513, S516, V518, Y520, Y523, F527	Q259, S260, V329, H331, A332, S333, T358, H361, E362, H365, E389, D393, E431, F435, F438, K489, F490, H491, N494, T496, Y498, Y501, F505
Perindoprilat	Q281, T282, H353, A354, S355, V380, H383, E384, H387, E411, D453, F457, F460, K511, F512, H513, S516, V518, Y520, Y523, F527	Q259, S260, V329, H331, A332, S333, T358, H361, E362, H365, E389, D393, E431, F435, F438, K489, F490, H491, N494, T496, Y498, Y501, F505
Quinaprilat	Q281, T282, H353, A354, S355, V379, V380, H383, E384, H387, E411, D453, F457, F460, K511, F512, H513, S516, V518, Y520, Y523, F527	Q259, S260, V329, H331, A332, S333, S357, T358, H361, E362, H365, E389, D393, E431, F435, F438, K489, F490, H491, N494, T496, Y498, Y501, F505
Fosinoprilat	Q281, T282, V351, H353, S355, W357, K368, V379, V380, H383, E384, H387, E411, D415, D453, K454, F457, F460, K511, F512, H513, S516, V518, Y520, Y523, F527, Q530	Q259, S260, V329, H331, S333, W335, K346, S357, T358, H361, E362, H365, E389, D393, E431, K432, F435, K489, F490, H491, N492, T494, Y498, Y501, F505, Q508
RXP407	Q281, V351, H353, S355, A356, W357, K368, V379, V380, H383, E384, H387, F391, H410, E403, E411, D453, F457, K511, F512, H513, S516, V518, Y520, R522, Y523, F527	Q259, V329, H331, S333, A334, W335, K346, S357, T358, H361, E362, H365, Y369, R381, H388, E389, E431, F435, K489, F490, H491, N494, T496, Y498, Y501, R500, F505
Omaparilat	Q281, K511, Y520, H353, H513, A354, H383, Y501	Q259, K489, Y498, H331, H491, A332, H361, Y501
Sampatrilat	H383, H387, E411, H513, Q281, K511, Y520, V380, H383, H387, F457, H513, Y523, F527, H353	H361, H365, E389, H491, Q259, K489, Y498, T358, H361, H365, F435, H491, Y501, F505, H331
FI	F375, H410, A356, R522, Y523, E411, V518, F512, H383, H387, Y523, K454, F457, F527, V380, H383	H388, Y369, F490, T358, H361, D393, S333, H388, F435, F490, T496, F505
FII	D415,H383,V380,F527,F457,K454,F512,V518,F391,N66,R522,W220,W357,Y62,E123,E403	H388, Y369, F490, T358, H361, D393, S333, H388, F435, F490, T496, F505
AD011012013	H383,H387,E411,E384,Y623,A356,E384,H353,S355,F512,V518,A354,H513,K511,Y520,Q281	H361,H365,E389,E362,Y601,A334,E362,H331,S333,F490,T496,A332,H491,K489,Y498,Q259

### 3.1 Classifications of ACE inhibitors

There are two classification methods for ACEs, which classify ACE according to the source of the ACE inhibitor and the molecular structure of the ACE inhibitor. Based on their sources, ACE inhibitors can be divided into two groups: ACE inhibitors from artificial chemical synthesis and ACE inhibitors from hydrolysis of natural products. ACE inhibitors are classified into three classes based on the molecular structure of the enzyme-binding sites to the active core of ACE: sulfhydryl-containing drugs, dicarboxylate-containing agents, and phosphonate-containing medicines.

### 3.2 Sulfhydryl-containing agents

#### 3.2.1 Captopril

Captopril was the first ACE inhibitor and was first synthesized by researchers from Bristol-Myers Squibb in 1975 (Smith). Because of its mechanism of action and development process, the discovery of captopril was considered a breakthrough. Captopril is an L-proline derivative, wherein L-proline is substituted on nitrogen with (2S)-2-methyl-3-thiolalkylpropionyl, which is a pyrrolidine monocarboxylic acid, an N-acylpyrrolidine, an alkanethiol and an L-proline derivative. Captopril is an N-thiokyl derivative of the alanine-proline dipeptide (Acharya et al., 2003). Captopril binds to sACE with a Ki1.4 nM affinity (Williams et al., 1996). The sulfhydryl of captopril binds with the divalent zinc ion of the active center of ACE in a variant tetrahedral geometrical configuration (Polakovičová and Jampílek, 2019). The carboxyl group of proline binds with conserved residues of ACE (Q281/Q259, K511/K489 and Y520/Y498) through hydrogen bonds and ionic bonds. The ketonic oxygen binds with two residues of ACE (H513/H491, H353/H331) through hydrogen bonding (Fernandez et al., 2001). Because of the small size of captopril, after captopril binds with either the ACE-N or the ACE-C, there are still vast residual spaces of substrate-binding sites. Captopril has been reported to have some N selectivity in connection with the concentration of chloride ions (Wei et al., 1992; Ehlers and Riordan, 2002). Captopril has an L-proline group, making it more bioavailable in oral formulations. In addition, captopril was found to have therapeutic effects other than cardiovascular effects. Although captopril can activate the Wnt signaling pathway, it can drastically reduce the expression of Wnt’s target genes, according to Riddiough et al.’s research Riddiough et al. (2021). These studies indicate that captopril may be helpful for tumor treatment. The intramolecular thiol moiety is linked to two serious side effects: hapten and immunological response. This immunological response, also known as agranulocytosis, could explain why some people get urticaria, severe stomachache, dyspnea, and swelling of the neck when they take captopril (Frohlich et al., 1984).

#### 3.2.2 Alacepril and zofenopril

##### 3.2.2.1 Alacepril

Alacepril is a variant of captopril. Alacepril can be considered an N-thiokyi derivative of the tripeptide alanine-proline-phenylalanine. Compared with captopril, alacepril has more phenylalanine, and the sulfhydryl of captopril is carbethoxylated. After entering the human body, alacepril is converted into desacetyl-alacepril by the acetylase. Desacetyl-alacepril is then converted into captopril to take effect. Compared with taking captopril, taking alacepril has a longer half-life period. Alacepril is clinically used in the treatment of hypertension. In addition, captopril was found to have therapeutic effects other than cardiovascular effects. Alacepril has been used in previous study to improve insulin resistance especially in patients with hypertension and diabetes mellitus (Yao et al., 2020).

##### 3.2.2.2 Zofenopril

Zofenopril is also a variant of captopril. Zofenopril is a proline derivative, i.e., 4-(phenylsulfanyl)-L-proline, in which the amine proton is replaced by a (2S)-3-(benzoylsulfanyl)-2-methylpropanoyl group (Remko et al., 2015). It is a thioester, an N-acyl-L-amino acid, an aryl sulfide and an L-proline derivative. Compared with captopril, the sulfhydryl of captopril is benzoylated, and the five-membered heterocycle is added with a thiophenol. Alacepril is a prodrug. Then, in the human body, alacepril is converted into desacetyl-alacepril by acetylase. Desacetyl-alacepril is then converted into captopril to take effect. Compared with taking captopril, taking alacepril has a longer half-life (Salvetti, 1990). Zofenopril is also a prodrug. After entering the human body, zofenopril is hydrolyzed into the active compound SQ26333. Compared with captopril, the active compound SQ26333 has a larger molecular weight and stronger lipophilic properties, meaning a stronger interaction with ACE and easier assimilation (Salvetti, 1990). Zofenopril has a clinical effect in the treatment of hypertension. In addition, zofenopril was found to have therapeutic effects other than cardiovascular effects. According to Macabrey et al.’s research, zofenopril had more favorable benefits in lowering SMC proliferation and restenosis than a nonsulfhydryl ACEI, even in normotensive animals, due to the production of hydrogen sulfide (Macabrey et al., 2021). These discoveries could have far-reaching clinical consequences for patients with vascular occlusive disorders including hypertension (Lv et al., 2021).

### 3.3 Dicarboxylate-containing agents

#### 3.3.1 Lisinopril and enalapril

##### 3.3.1.1 Lisinopril

**FIGURE 2:**
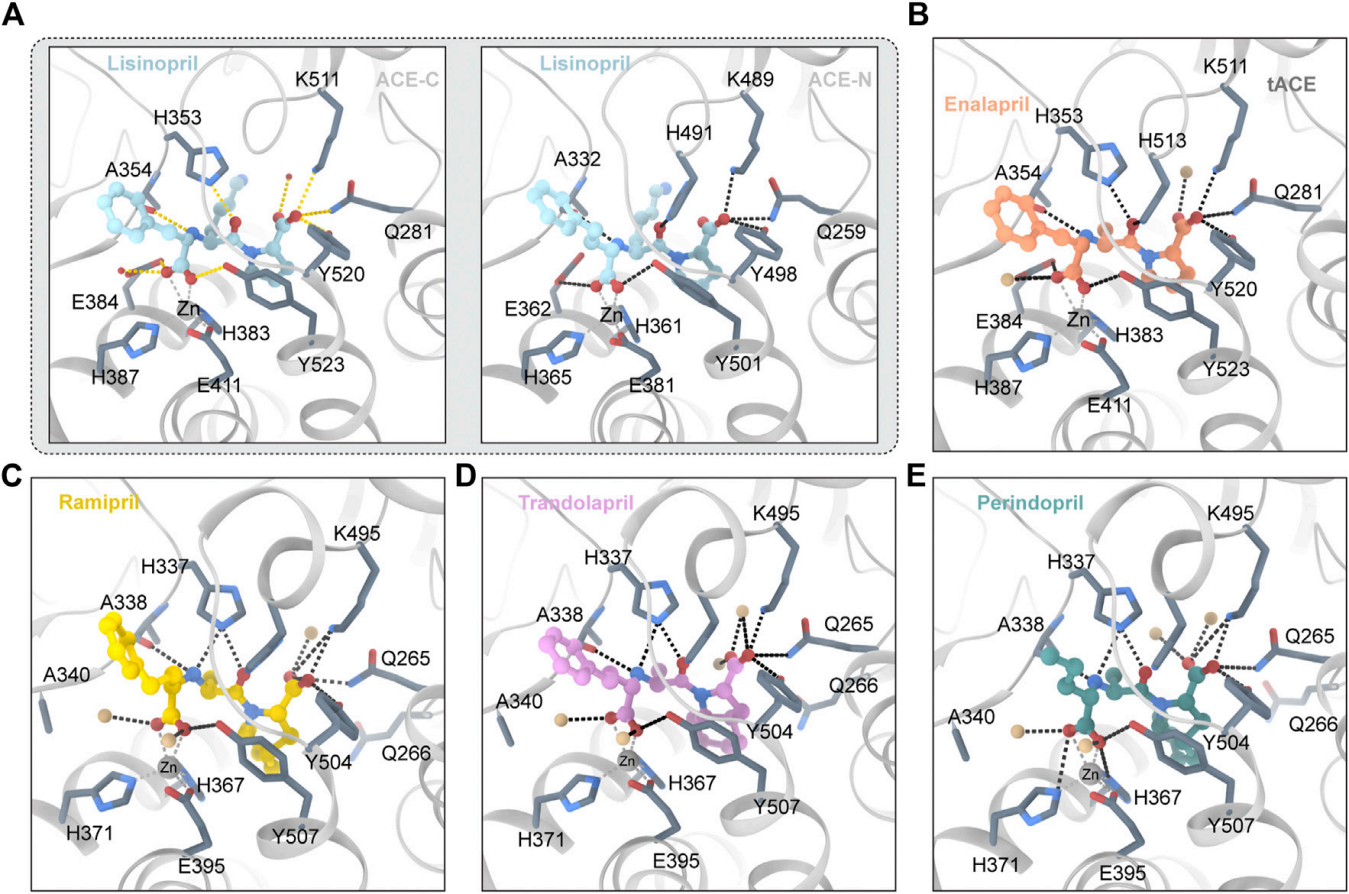
**(A)** Lisinopril and ACE (PDB ID:1O86 2C6N) Critical residues are identified using a one-letter code. The ligand molecule is shown in blue by balls and sticks Hydrogen bonds are represented by dashed lines. **(B)** Enalapril and ACE (PDB ID:1UZE) The ligand molecule is shown in orange by balls and sticks. **(C)** Ramipril and ACE (PDB ID:2X92) The ligand molecule is shown in yellow by balls and sticks. **(D)** Trandolapril and ACE (PDB ID:2X93) The ligand molecule is shown in pink by balls and sticks. **(E)** Perindopril and ACE (PDB ID:2X94) The ligand molecule is shown in green by balls and sticks.

Lisinopril is a derivative of the tripeptide phenylalanine-lysine-proline. Lisinopril binds to ACE with Ki ≈ 0.39 nM (Wei et al., 1991). Lisinopril is adjacent to the HExxH motif and the divalent zinc ion (Tzakos and Gerothanassis, 2005). The crystal structure of lisinopril and ACE is shown in Figure 2A. The crystal structure of Lisinopril and ACE-C binding shows that isinopril binds ACE in a highly ordered and extended conformation (Natesh et al., 2003). The carboxyl of phenylpropyl binds with the divalent zinc ion and forms a hydrogen bond with the carboxylate of the side chain of E384 and the hydroxyl of the side chain of Y283 (Akif et al., 2012). The lysine part interacts with the S1′ subsite and forms a salt bridge with E162 and D377 (Tzakos et al., 2003; Polakovičová and Jampílek, 2019). The proline part interacts with the S2′ subsite through hydrophobic interactions with the aromatic nucleus and hydrogen bonds with K511, Q281 and Y520 (Tzakos and Gerothanassis, 2005). Despite the structural homology between ACE-C and ACE-N, there are some significant differences between the active sites. E162 in the S1 ‘subunit of ACE-C is replaced by D140 in ACE-N, but D140 cannot make contact with lisinopril (Corradi et al., 2006). D377 in the S1 ‘subunit of ACE-C is replaced by Q355 in ACE-N; however, Q355 is distant from lisinopril and does not directly interact with lisinopril (Corradi et al., 2006). These differences may explain why Lisinopril has a higher ACE-C selectivity. Lisinopril is an ACEI and considered as the gold standard drug for the treatment of hypertension (James et al., 2014). High blood pressure is usually treated first (James et al., 2014). Lisinopril is taken orally and can take up to 4 weeks to fully take effect. In addition to being used to treat cardiovascular disease, lisinopril has been found to have other effects in recent years. The researchers reported a series of cases in which patients had a delayed inflammatory response after receiving the COVID-19 vaccine (Munavalli et al., 2021). Using lisinopril rapidly improved their conditions by inhibiting angiotensin-converting enzyme (ACE). According to Fischer et al.’s research, ACEI use is associated with a lower risk of developing clinically recognized leiomyoma in adult hypertensive women (Fischer et al., 2021; Firouzabadi et al., 2022). Researchers discovered that perioperative ACEI use may be a previously unknown risk factor for surgical site infection (Trikha et al., 2020).

##### 3.3.1.2 Enalapril

Enalapril is ethyl 4-phenylbutanoate in which an amino group of L-alanyl-L-proline replaces a hydrogen alpha to the carboxy group (S-configuration) (Kumar et al., 2010). Enalapril is a dipeptide and a dicarboxylic acid monoester (Weller et al., 1984). Enalapril is converted into enalaprilat by acetylase. Enalaprilat is a derivative of the tripeptide phenylalanine-alanine-proline. The crystal structure of enalapril and ACE is shown in Figure 2B. Under conditions of different concentrations of chloride ions, enalaprilat’s affinities for the ACE-N and ACE-C domains are slightly different (Tzakos et al., 2003; Bernstein et al., 2013). At high chloride concentrations, enalaprilat prefers to inhibit the ACE-N domain. At low chloride concentrations, however, enalaprilat prefers to inhibit the ACE-C domain (Wei et al., 1991). Enalapril preferentially inhibits the ACE-N domain but not the ACE-C domain at low chloride concentrations (Natesh et al., 2004). This may be because chloride ions may induce conformational changes in ACE (Natesh et al., 2004). The crystal structure of enalapril combined with the ACE-C domain indicates that enalapril lacks contact with the S1 ‘pocket of ACE-C compared with lisinopril (Natesh et al., 2004). This may explain why Lisinopril and ACE-C have a higher affinity than enalapril and ACE-C (Natesh et al., 2004). Enalapril is bound by the carboxyl group and the catalytic core divalent zinc ion, with the amino terminal phenyl moieties of enalapril in a hydrophobic pocket consisting of hydrophobic residues such as F512 and V518 (Natesh et al., 2004). The enalaprilat P1’s group is different from that of lisinopril. Enalaprilat’s P1′ group is an alanyl group, and lisinopril’s group is a lysyl group. The alanyl group replacing the lysyl group leads to a lower affinity for the ACE-C domain and a higher affinity for the ACE-N domain (Tzakos and Gerothanassis, 2005). Enalapril is used in the treatment of hypertension, diabetic nephropathy, and heart failure (Barnett, 2005). It is typically used in conjunction with diuretics such as furosemide to treat heart failure. It is administered either orally or intravenously. After oral administration, it usually takes effect within an hour and lasts for 1 day. In addition, enalapril was found to have other effects. Yang et al’s research shows for the first time that enalapril increases the sensitivity of CRC cells to 5-FU (Yang Y. et al., 2020). Borchert’s research shows that early angiotensin-converting enzyme inhibitor treatment only reduces acute cardiac and neuroinflammation (Borchert et al., 2020).

#### 3.3.2 The variants of lisinopril and enalapril

**FIGURE 3:**
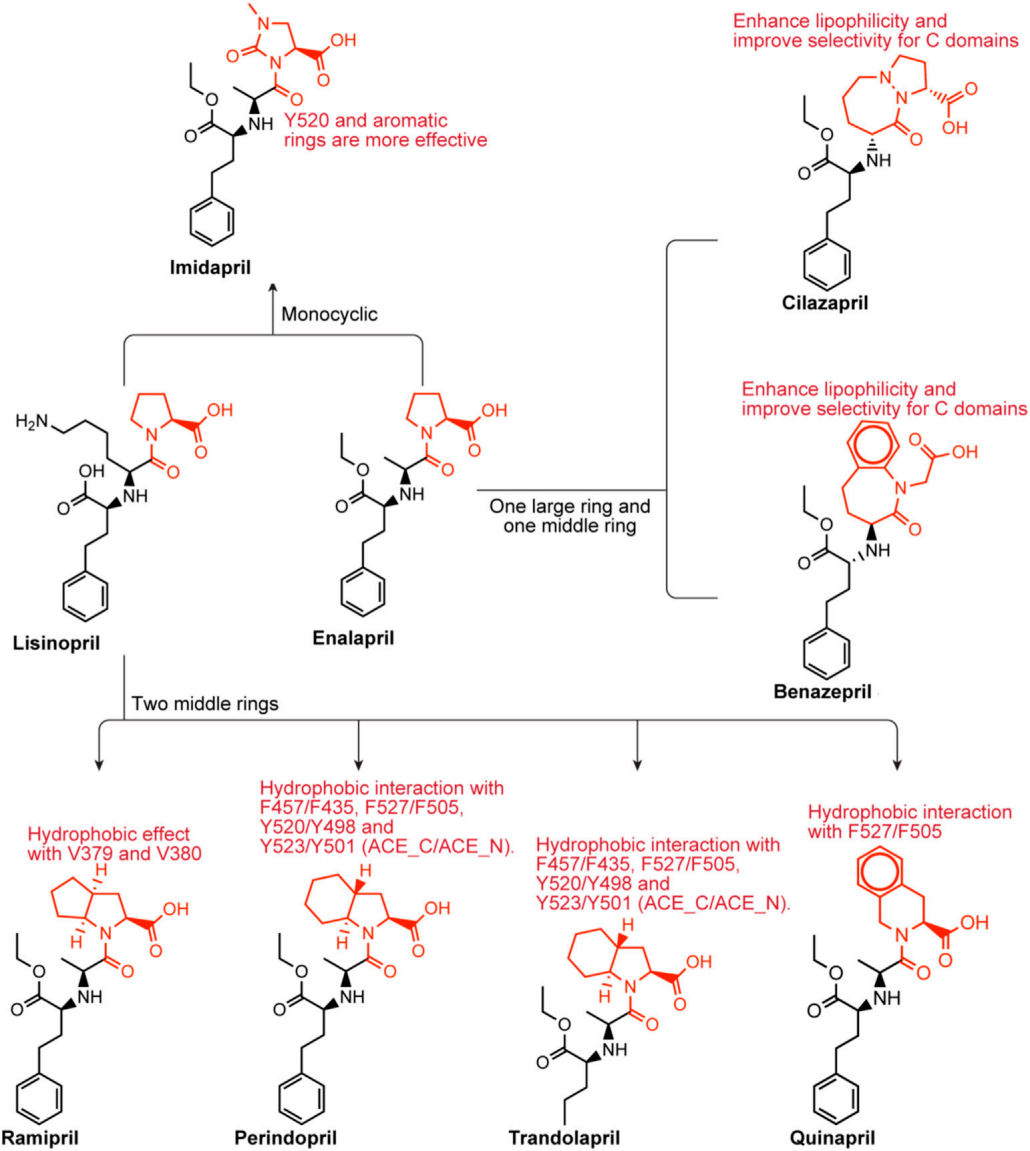
Chemical structures of dicarboxylate-containing agents. The differences of chemical structures are in red.

Lisinopril and enalapril are first-generation ACE inhibitors. Researchers have developed some new ACE inhibitors by chemically modifying the tetrahydropyrrole ring of proline. According to the chemically modified structure, new ACEI can be separated into three groups: a five-membered ring monocyclic group, a bicyclic group with two middle rings, a bicyclic group with one middle ring and one large ring (Figure 3).

##### 3.3.2.1 Imidapril

Imidapril belongs to the imidazolidine class, which is (4S)-1-methyl-2-oxoimidazolidine-4-carboxylic acid with the hydrogen of the imidazolidine nitrogen replaced by the (1S)-1-[(2S)-1-ethoxy-1-oxo-4-phenylbutan-2-yl]aminoethyl group (Harahap et al., 2021). Imidapril is a precursor to the ACEI imidaprilat, used to treat chronic heart failure. Imidapril is a variant of enalapril. Different from enalapril, the tetrahydrogpyrrole ring of proline is replaced by the dihydrogimidazole ring, which may lead to a stronger interaction with Y520s aromatic nucleus.

##### 3.3.2.2 Ramipril perindopril trandolapril quinapril

###### Ramipril

Ramipril is a dipeptide that serves as a precursor to ramiprilat, the active metabolite produced by ethyl ester hydrolysis. Because there is currently no crystal structure of ramipril bound to human ACE, we chose the crystal structure of ramipril bound to AnCE (the ACE analogue in Drosophila) (in the Figure 2C). Ramipril is similar to enalapril in the structure, only adding a cyclopentane ring to the tetrahygropyrrrole ring. Compared with enalapril, ramipril has a better affinity for the ACE-C domain because the P1′ and P2′ groups have an additional hydrophobic interaction with V379 and V380 in the S1′ and S2′ subsites (Tzakos and Gerothanassis, 2005). Ramipril is an ACEI commonly used to treat especially people over 55 hypertension, heart failure, and diabetic nephropathy (Hanif et al., 2010). Ramipril is administered orally. Researchers developed self-nanoemulsifying ramipril tablets to enhance drug dissolution and stability (Alhasani et al., 2019). In addition to being used to treat cardiovascular disease, ramipril has been found to have other effects. According to Wu’s study, ramipril provides sustained survival benefits for patients with post-AMI clinically defined heart failure (Wu J. et al., 2021). Tan found that the ACE inhibitor ramipril suppressed the TGF-1/Smad and TGF-1/TAK1 pathways in scar formation by mediating downstream peptides (Tan et al., 2018). These findings suggest that using ACEIs to inhibit both Smad and TAK1 signaling could be a good strategy for developing new antifibrotic drugs.

###### Perindopril and trandolapril

Because there is currently no crystal structure of perindopril bound to human ACE, we chose the crystal structure of perindopril bound to AnCE (the ACE analogue in Drosophila) (in the Figure 2D). Compared with ramipril, perindopril adds a cyclohexane ring to the tetrahydropyrrole ring and has an aliphatic chain in the P1 group instead of an aromatic nucleus. In ACE C/ACE N, alkyl groups occupy the S1 enzyme subunits represented by the common residues S355/S333, F512/F490 (forming hydrophobic interactions), V518/T496 and S516/N494 (Tzakos and Gerothanassis, 2005). Furthermore, the perhydroindole ring system interacts with the hydrophobic plaques formed by the aromatic rings of F457/F435, F527/F505, Y520/Y498, and Y523/Y501 (ACE C/ACE N) (Tzakos and Gerothanassis, 2005). The V379/S357 mutation, like ramiprilat, provides an additional hydrophobic interaction with the S2′ subordination in the C domain relative to the associated subunit in the N domain which could explain the 40-fold increase in perindopril selectivity (Tzakos and Gerothanassis, 2005). In addition to being used to treat cardiovascular disease, perindopril has been found to have other effects. A research by Barutta showed that the combination AM6545 and perindopril can reverse experimental diabetic nephropathy, possibly because combination therapy has the ability to favor macrophage polarization toward an M2 macrophage phenotype (Barutta et al., 2018; Moratal et al., 2021).

Trandolapril is a heterobicylic compound that is (2S,3aR,7aS)-1-[(2S)-2-aminopropanoyl]octahydro-1H-indole-2-carboxylic acid, with the amino group’s hydrogen substituted by a (2R)-1-ethoxy-1-oxo-4-phenylbutan-2-yl group (Pal Khaket et al., 2012). Because there is currently no crystal structure for qundopril bound to human ACE, we chose the crystal structure for qundopril bound to AnCE (the ACE analogue in Drosophila) (in the Figure 2E). Qundopril can be seen as an intermediate variant of perindopril and ramipril. Trandolapril and perindopril have side effects similar to those of other ACE inhibitors.

###### Quinapril

Quinapril belongs to the isoquinoline class, and its chemical formula is (3S)-2-L-alanyl-1,2,3,4-tetrahydroisoquinoline-3-carboxylic acid, in which the alpha-amino group of the alanyl residue has been replaced by a 1-ethoxycarbonyl-4-phenylbutan-2-yl group (the all-S isomer). It is a member of the isoquinolines, a dicarboxylic acid monoester, an ethyl ester and a tertiary carboxamide. Quinapril is similar to drugs with the same type of structure. The difference is that it is a tetrahydroisoquinoline ring. The tetrahydroisoquinoline ring leads to a difference in P2′ group. The aromatic rings of this group, such as in ramipril and perindopril enhance lipophilicity in the molecule (Tzakos and Gerothanassis, 2005). Quinapril has a strong C domain selectivity. This may be due to the additional hydrophobic effect between the S1′ and S2′ subunits of the C domain and the P1′ and P2′ of quinapril (Tzakos and Gerothanassis, 2005). Weak N domain inhibition may be attributed to unproductive regulation of the P1 group at the S1 subsite (Tzakos and Gerothanassis, 2005). Quinapril is usually used as the hydrochloride to treat hypertension and congestive heart failure (Townend et al., 1992).

##### 3.3.2.3 Benazepril cilazapril

###### Benazepril

Benazepril has a role as an EC 3.4.15.1 (peptidyl-dipeptidase A) inhibitor and a prodrug. Benazepril is a benzazepine, a dicarboxylic acid monoester, an ethyl ester and a lactam. Benazepril resembles quinapril in structure. They have a bulkier bicyclic group in the P2′ group. They may have a higher C selectivity. The reason is similar to quinapril’s. Benazepril is a blood pressure medication that can be used to treat heart failure and diabetic kidney disease (Fan et al., 2006). Tiredness, dizziness, coughing, and light-headedness when standing are common side effects. Use during pregnancy may be harmful to the baby, but use while breastfeeding may be safe.

###### Cilazapril

Cilazapril is a kind of ACEI. Cilazapril is a prodrug that is hydrolyzed after absorption to its main metabolite cilazaprilat. Cilazapril is an ethyl ester, a pyridazinodiazepine and a dicarboxylic acid monoester. Cilazapril resembles quinapril in the structure. They have a bulkier bicyclic group in the P2′ group. They may have a higher C selectivity. The reason is similar to quinapril’s. Cilazapril which can be used to treat hypertension and heart failure has similar side effects to other ACE inhibitors.

### 3.4 Phosphonate-containing agents

#### 3.4.1 Fosinopril

**FIGURE 4:**
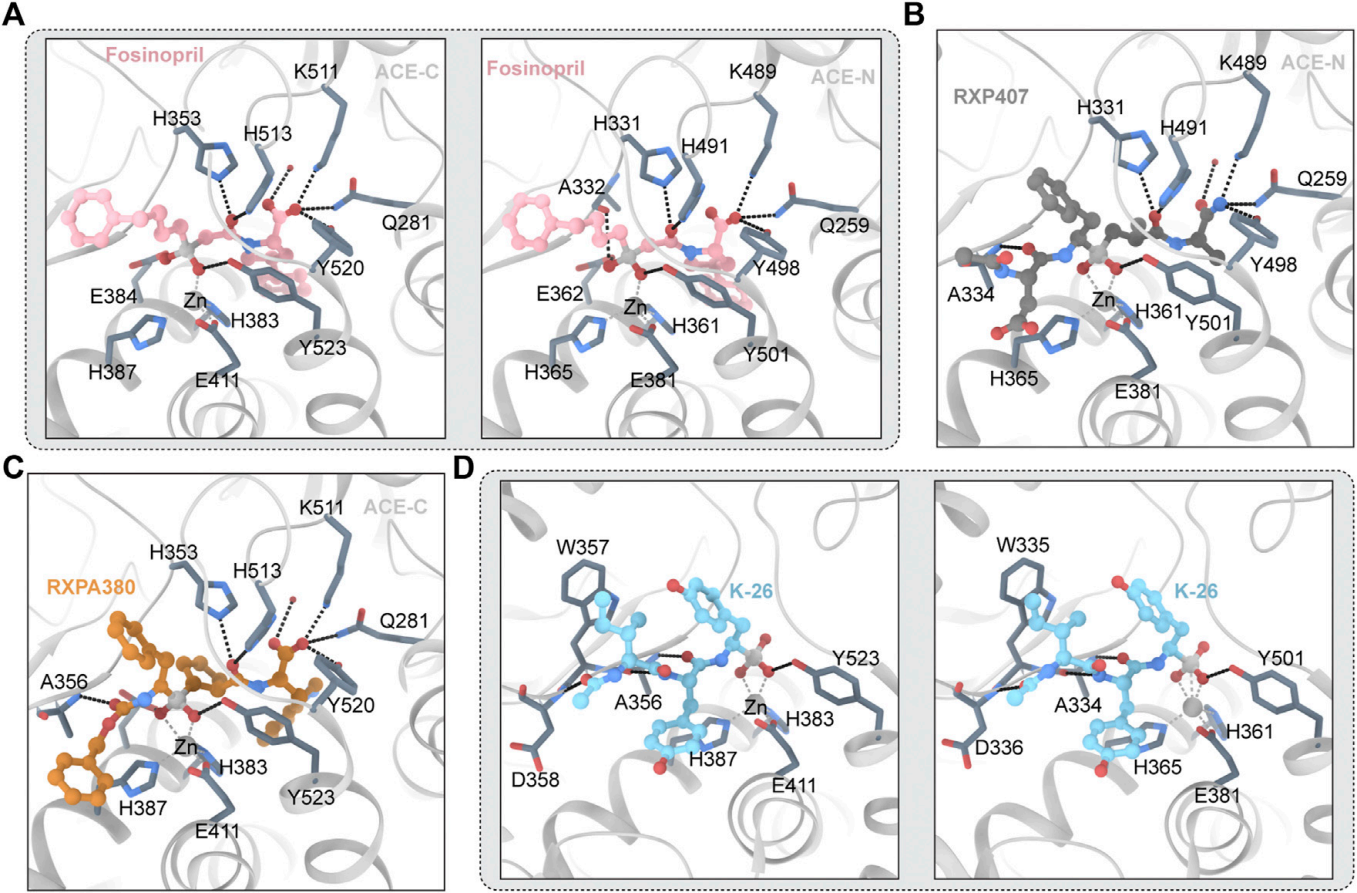
**(A)** Fosinopril and ACE (PDB ID:7Z70 7Z6Z) The ligand molecule is shown in pink by balls and sticks. **(B)** RXP407 and ACE (PDB ID:3NXQ) The ligand molecule is shown in dark blue by balls and sticks. **(C)** RXPA 380 and ACE (PDB ID:2OC2) The ligand molecule is shown in orange by balls and sticks. **(D)** K-26 and ACE (PDB ID:4BZR 4BZS) The ligand molecule is shown in vlue by balls and sticks.

Fosinopril is commercially available phosphonate-containing ACEI. Fosinopril is a N-acyl derivative of (4S)-cyclohexyl-L-proline that contains a phosphinate ester (Panday, 2011). Fosinopril is a prodrug that is hydrolyzed to metabolite phosphininc acid fosinopril (Hui et al., 1991). The phosphonate of fosinopril binds with the divalent zinc ion of ACE. The Ki values that inhibit the hydrolysis of different substrates vary significantly (Tzakos and Gerothanassis, 2005). The crystal structures of fosinopril and ACE-C and ACE-N (Figure 4A) show that P1 and P2 occupy the non-primary subunit, and P1 ‘and P2′ occupy the primary subunit (Cozier et al., 2022). Fosinopril forms an extensive interaction network with amino acid residues in S1, S2, S1 ‘and S2′, and all hydrogen bonds and electrostatic interactions between ACE and fosinopril are conserved in ACE-C and ACE-N (Cozier et al., 2022). Fosinopril is bound by the phosphonate group and the catalytic center divalent zinc ion. One oxygen atom in the phosphine group interacts with Y501/Y523 (ACE-N/ACE-C) and the other oxygen atom interacts with E362/E384 (ACE-N/ACE-C) (Cozier et al., 2022). The P1 'backbone oxygen interacts with H331/H353 and H491/H513 (ACE-N/ACE-C) through hydrogen bonds, and the carboxyl group at the end of P2′ directly interacts with Q259/Q281, K489/K511 and Y498/Y520 (ACE-N/ACE-C) (Cozier et al., 2022). Most hydrophobic interactions are also conserved in ACE-C and ACE-N. The P1 carbon chain and P2 phenyl groups were extended along the hydrophobic butyl consisting of H331/H353, S333/S355, W335/W357, F490/F512 and T496/V518 (ACE-N/ACE-C) (Cozier et al., 2022). The backbone carbon of P2 'interacts with H491/H513 and Y501/Y523 (ACE-N/ACE-C), while the side chain of P2′ pyrrolidine forms hydrophobic interactions with H361/H383, Y501/Y523 and F435/F457 (ACE-N/ACE-C) (Cozier et al., 2022). The cyclohexane ring forms hydrophobic interactions with S357/V379, T358/V380, H361/H383 and F505/F527 (ACE-N/ACE-C) (Cozier et al., 2022). Although the interaction between ACE and fosinopril is conserved in both ACE-N and ACE-C, single site mutations in T496 in ACE-N and V518 in ACE-C lead to changes in hydrophobicity of the S1 subunit, which in turn leads to changes in the environment and orientation of the P2 'side chain (Cozier et al., 2022). Finally, fosinopril had high ACE-C selectivity. In addition, in ACE-N, the cyclohexane and pyrrolidine rings are perpendicular, and in ACE-C, the cyclohexane and pyrrolidine rings are parallel (Cozier et al., 2022). The researchers believe that the unique V379 and V380 residues in ACE-C will confer selectivity to the fosinopril C domain through hydrophobic interactions with the cyclohexane ring (Cozier et al., 2022). The medication was developed specifically for use in patients with renal impairment to treat hypertension through manipulation of metabolism and excretion, which distinguishes it from other members of the ACE inhibitor drug class (Marin et al., 2001). The hepatobiliary pathway clears 50% of the drug, which helps to compensate for decreased renal clearance (Sica et al., 1991). The remaining 50% is excreted in the urine. There is no need to change the dosage.

##### 3.4.1.1 RXP407 and RXPA380

RXP407 is an inhibitor of the ACE-N domain developed by Dive et al., in 1999 that is highly selective. The crystal structure of RXP407 and ACE is shown in Figure 4B. It shows 3 orders of magnitude ACE-N domain selectivity to the ACE-C domain and is expected to become a drug for the treatment of fibrosis (Bernstein et al., 2013). However, the insufficient performance of ADME makes it an inferior drug. RXP407 has a polypeptide-like structure. RXP407 is bound by phosphate groups and divalent zinc ions. The interaction of acidic aspartic acid at the P2 position with R381 and Y369 on the S2 subunit was critical for the compound’s N domain selectivity, according to an analysis of the crystal complex formed by the N domain with the inhibitor.

RXPA380 is a high selectivity ACE-C domain inhibitors (Corradi et al., 2007). Figure 4C depicts the crystal structure of RXPA380 and ACE. RXPA380 and the ACE-C domain have a stronger direct interaction than other nonselective ACE inhibitors, possibly because RXPA380 has a more complex structure. The aromatic interaction between benzyl acetate and F391 at the P2 position in the S2 subunit is thought to be responsible for RXPA380s selectivity to the ACE-C domain (Kröger et al., 2009). In addition, the massive tryptophan at the P2′ position in RXPA380 and V 80 and V379 have hydrophobic effects, and these two residues are substituted by S3 358 and T358 in the ACE-N domain (Akif et al., 2010).

##### 3.4.1.2 K-26

K-26 adopts a similar conformation in both domains, retaining nearly identical potential hydrogen bond networks (Kramer et al., 2014). Figure 4D depicts the crystal structure of K-26 and ACE. The S1 binding pocket contains phosphate coordination zinc and AHEP side chains, the S2 binding pocket contains tyrosine side chains, and the S3 binding pocket contains ACE-Ntyl isoleucine (Kramer et al., 2014). The AHEP side chain’s aromatic group may form a hydrophobic interaction with V518 (T496 in the ACE-N domain). More water molecules that interact with K-26 can be found in the ACE1-C domain. The tyrosine side chain in the S2 binding pocket may form a hydrophobic interaction with F391 and form a conserved aromatic accumulation effect with H387 and H410 (Kramer et al., 2014). The hydrogen bond between the skeletons of tyrosine and A356 (A334 in ACE-N) is also visible in K-26. Furthermore, water interacts with the hydroxyl groups of K-26’s tyrosine side chain and the skeleton’s R 402 and G404 residues which are highly similar in the two domains of the ACE, with the exception of E403. R381’s long polarity sidechains may block these water-mediated interactions in the N-domain which leads to reducing affinity with K-26 (Kramer et al., 2014). It has been shown by previous research that amino acid side chains occupy the S1 and S2 binding pockets of ACE, influencing the domain’s selectivity. The S3 binding pocket are unknown regions for inhibitor design. Synthetic analogs can be concentrated in the S3 position. The crystal structure suggests that the hydrophobic interaction of the S2 bit and the interaction of water with the S2 and ACE-Ntyl groups provide the preferential C-domain selectivity observed by K-26 (Kramer et al., 2014).

### 3.5 Dual target inhibitor

#### 3.5.1 Omapatrilat

**FIGURE 5:**
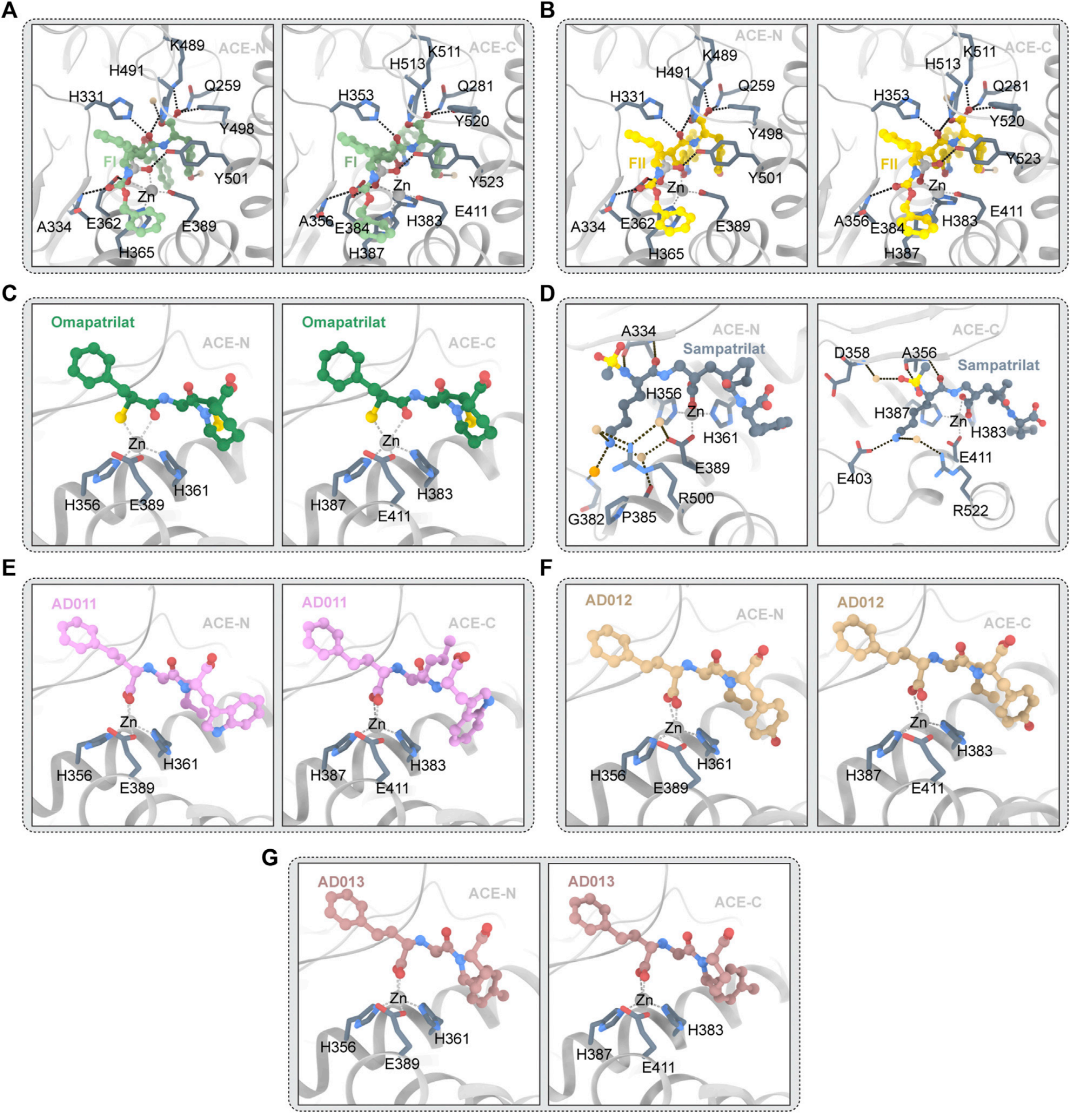
**(A)** FI and ACE (PDB ID:4CA5 4CA6) The ligand molecule is shown in green by balls and sticks. **(B)** FII and ACE (PDB ID:2XY9 2XYD) The ligand molecule is shown in yellow by balls and sticks. **(C)** Omapatrilat and ACE (PDB ID:6H5W 6H5X) The ligand molecule is shown in dark green by balls and sticks. **(D)** Sampatrilat and ACE (PDB ID:6F9T 6F9V) The ligand molecule is shown in grey by balls and sticks. **(E)** AD011 and ACE (PDB ID:7Q24 7Q27) The ligand molecule is shown in pink by balls and sticks. **(F)** AD012 and ACE (PDB ID:7Q25 7Q28) The ligand molecule is shown in brown by balls and sticks. **(G)** AD013 and ACE (PDB ID:7Q26 7Q29) The ligand molecule is shown in red by balls and sticks.

Omapatrilat is a dual inhibitor of ACE and NEP (Chen et al., 2019). Figure 5C depicts the structure of omapatrilat and ACE. Omapatrilat is a tripeptide mimic with a carboxylic acid “C-terminus,” a bicyclic group which occupies the S2′ and S1′ subunits, and a sulfur-containing phenylalanine analog which interacts with zinc ions (Cozier et al., 2018a). “C-terminal” carboxylic acids are tightly bound, because the carbonyl oxygen atoms in the carboxyl group form hydrogen bonds with Q259, K489 and Y498 (Q281, K511 and Y520 in ACE-C), and the oxygen atoms of the hydroxyl group of the carboxyl group interact with K489/511 (Cozier et al., 2018a; Chen et al., 2019). The side chains of H331 and H491 of ACE-N, as well as the skeleton of A332 of ACE-N, interact with the backbone’s bicyclic omapatrilat group (H353 H513 and A354 of ACE-C) (Tzakos and Gerothanassis, 2005). The carbonyloxy atoms in omapatrilat’s amide group interact with H361 and Y501 of ACE-N (H383 and Y501 of ACE-C) and form a bidentate interaction with Zn2+ with S2 (Cozier et al., 2018a). It was observed that the high affinity and slow shedding rate of omapatrilat may be due to strong bidental interactions with zinc ions (Cozier et al., 2018a).

#### 3.5.2 Sampatrilat

Semprilla is a dual inhibitor of angiopeptidase or angiotensin-converting enzyme and neutral endopeptidase with the potential to provide greater benefits than conventional ACEs (Sharma et al., 2016). Figure 5D depicts the crystal structure of sampatrilat and ACE. In ACE-C domain, the zinc ions are centered in a high affinity surrounding of H383, H387, and E411 (H361, H365, and E389 in ACE-N), and sampatrilat’s carboxyl groups bind to zinc ions (Cozier et al., 2018b). In ACE-C and ACE-N, the P1ʹ backbone carbonyl groups bind to H513 and H491, respectively. ACE-C Q281, K11, and Y520 (Q259, K489, and Y498 of ACE-N) all interact with P2ʹ-terminal carboxylic acids (Cozier et al., 2018b). In addition, ACE-C residues V380, H383, H387, F457, H513, Y523, and F527 (T358, H361, H365, F435, H491, Y501, and F505) interact with many hydrophobic interactions of the P1ʹ and P2ʹ groups in ACE-N and are also conserved (Tzakos and Gerothanassis, 2005; Cozier et al., 2018b). Structural differences between the S1ʹ and S2ʹ subitems relate to water-mediated interactions to a large extent. Two water molecules are near the P2 tyrosine group in the ACE-N complex structure and the Sampatrilat-ACE-C complex structure. However, changes in the protein residues involved in these water-mediated interactions occur as a result of small movements of inhibitors and water positions (Cozier et al., 2018b). There are no water molecules near the tyrosine inhibitor group in the SamAsp-ACE-C complex. The orientation of the H353 side chain in the sampatrilat-ACE-C structure is another difference between these structures (Cozier et al., 2018b). In the other three structures, this residue (H331 in ACE-N) interacts with the P1ʹ backbone and main chain carbonyl groups of the inhibitor to form hydrogen bonds and hydrophobic interactions, respectively. H353 is oriented differently in the sampatrilat-ACE-C structure, and polyethylene glycol molecules are bound in the free space (Cozier et al., 2018b). This mimics the hydrophobic interactions observed in other structures and results in water-mediated interactions with the sampatrilat terminal carboxylates (Cozier et al., 2018b).

#### 3.5.3 FI and FII

##### 3.5.3.1 FII

FII is a dual inhibitor of vascular peptidase (also known as angiotensin-converting enzyme) and neutral endopeptase (Sharma et al., 2020). The crystal structure of FII and ACE is shown in Figure 5B. FII binds to ACE-C differently from other inhibitors, with two molecules binding OFFI and one molecule binding ACE. One molecule (hereafter referred to as FII-A) binds to the S1, S2, S2′ sites of the expected ACE-C active site, while an unexpected second molecule (hereafter referred to as FII-B) forms an intermolecular aromatic (pi-pi) interaction with FII-A (Akif et al., 2011). FII-A’s phosphoyl-zinc-binding group interacts with the divalent zinc ion (Perini et al., 2020). The localization of FII-A in the active site of ACE-C is significantly different from that of lisinopril, etc., more specifically, the localization of zinc phosphorylated groups and inhibitor molecules in P1, P1′, and P2′ (Akif et al., 2011). In P1′, the nitrogen atoms of the isoxazole ring interact with D415. The isoxazole ring’s oxygen atoms appear to interact directly with H383, the active central residue (Akif et al., 2011). The large side chain position of FII at P1 extends into the S2′ pocket in an unusual direction. The hydrophobic interaction provided by V380 may have stabilized this particular orientation (Akif et al., 2011). However, it is also possible that the unique topology of the ACE subsites S1′ and S2′ forms a very large cavity, wide enough to accommodate the side chain of the massive P1′ S or R configuration (Akif et al., 2011). The orientation of the P1′ side chain may be similar to the orientation of the lysine residue in lisinopril in the S configuration (Galanis et al., 2004). The aromatic residues F527 and F457 maintain the inhibitor P2′C-terminal tyrosine side chain in a hydrophobic interaction, which differs slightly from the orientation of RXPA380s tryptophan side chain (Akif et al., 2011). Furthermore, hydrogen bonds with D415, H383, and K454 anchor the hydroxyl group of the inhibitor tyrosine moiety (Akif et al., 2011). The hydrophobic interaction provided by F512 and V518 holds the side chain at the P1 position (Akif et al., 2011). Finally, as observed in RXPA380, the phenyl at the dual inhibitor P2 position has an aromatic contact with F391. In spite of this unusual binding pattern, FII-A has 16 hydrogen bonds with protein atoms, including 7 water-mediated hydrogen bonds, and several atoms of FII-A interact with 10 protein side chains (Akif et al., 2011). Nine hydrogen bonds and two direct interactions with N066 and R522 make up the FII-B-ACE-C interaction. The isoxazole phenyl group of FII-B and the aminobenzoyl group of the inhibitor FII-A have a strong aromatic (pi-pi) accumulation effect in the host position. The P1 phenyl of the inhibitor FII-B is involved in interaction with the residue W220 (Akif et al., 2011). The isoxazolium phenyl in FII-B interacts aromatically with W357. Y62 seems to have hydrophobic effects on the tyrosine group at the P2′ position of FII-B (Akif et al., 2011). The phosphine’s oxygen atom seems to interact directly with E123 and E403, but only weakly with K118 (Akif et al., 2011).

No clear two-molecule FII or one-molecule ACE-N binding events were found in the FII and ACE-N interactions. The P2-phenyl group of FII is controlled by H388- and Y369-mediated hydrophobic interactions (Akif et al., 2011). F490 appears to interact with the second phenyl at the P1 position of FII. The isoxazole ring’s nitrogen atom appears to play a role in interacting with T358, while the large-volume isoxazole ring interacts directly with the Nd1 atom of H361 at the P1′ position (Akif et al., 2011). The hydroxyl group of the tyrosine fraction in the P1 group of FII interacts with D393’s OD2 atom directly (Sharma et al., 2016). Additionally, inhibitors have hydrophobic effects on S333, H388, F435, F490, T496, and F505 (Akif et al., 2011). A comparison of the active sites of ACE-N and C-FII-B ACE’s binding site reveals the presence of several key residues that prevent FII binding (Arendse et al., 2022). In the ACE-N active site, L32, S35, V36, W203, and R381 were replaced with W59, Y62, A063, M223 and E403 in the ACE-C, respectively (Akif et al., 2011).

##### 3.5.3.2 FI

ACE/ECE1 FI dual inhibitors are competitive inhibitors of ACE-N and ACE-C. Figure 5A depicts the crystal structure of FI and ACE. The selectivity for ACE-C is approximately 440 times. FI, like FII, binds to all four substrate subsites. FI’s two phosphine oxygen atoms are directly coordinated with the active center’s catalytic zinc ions. Furthermore, it is made up of 12 hydrogen bonds, four of which are mediated by the water molecule, according to HBPLUS′ calculations (Masuyer et al., 2014). Aromatic interactions with F375 and H410, as well as hydrogen bonds between the carbonyl oxygen and A356 backbone nitrogen atoms, keep the phenyl group at the P2 position of the inhibitors (Masuyer et al., 2014). Water molecules bind to R522, Y523 and E411 through the backbone amide nitrogen, as seen in the FII complex (Masuyer et al., 2014). The hydrophobic interactions with the residues V518 and F512 keep the second phenyl group of FI at the P1 position (Masuyer et al., 2014). Direct hydrogen bonding with the hydroxyl groups of H383, H387, and Y523 further anchors the phosphine oxygen atoms of FI (Masuyer et al., 2014). Through the interaction of water molecules between its OH atoms and K454, the P2′C-terminal tyrosine portion of FI seems to interact with the residues F457 and F527 through hydrophobic interactions (Masuyer et al., 2014). In the S conformation of FI, the thick side chain at P1′ is clearly visible. The trunk of V380 seems to form a water-mediated bond with isoxazolidyl. It is surprising that isoxazolidines have a similar orientation in FII. The R configuration in FII keeps the group closer to the catalytic site, allowing direct hydrogen bonding with H383 (Masuyer et al., 2014). The whole binding mechanism resembles that of other phosphonium inhibitors, but there are significant differences at the P1′ site. The secondary binding site in ACE-C is a special feature of FII which is accomplished through hydrophobic interactions with the ACE-C allosteric site (Masuyer et al., 2014). This phenomenon is not seen in FI. Despite the fact that FI and FII have similar P2 configurations, FI’s S orientation prevents accumulation interactions because secondary molecules clash with inhibitors of the primary binding site. However, this significant difference does not result in a higher KI for FII which implies that FII binding at the secondary site is a synchronous event which requires binding inhibitors at the primary site (Masuyer et al., 2014). More binding experiments will reveal the significance of allosteric binding sites for FII and aid in the development of a new inhibitor that targets this region with the goal of preventing substrate binding. However, in this case, the suppression of the catalytic efficiency may depend on the main site.

The overall binding mode of FI and N_ACE is similar to the binding mode described by FII, and the tripeptide backbones of FI and FII overlap well (Masuyer et al., 2014). The catalytic coordination of zinc ions with FI’s phosphine oxygen atoms is the main anchor. Ten hydrogen bonds provide additional binding strength. The P1′ position is the only difference between the two stereoisomers combined, as expected. Due to the side chain rotation of 180°, FI’s isoxazoliumyl group does not appear to allow weak hydrogen bonds to form between FII and T358. The P1′ huge side chain fits into the large ACE-N S1′ cavity but appears to interact with proteins limitedly which forms a stacked arrangement with tyrosine groups at P2′, which themselves are encircled by aromatic residues and form anchor points whose C-terminal oxygen atoms bind strongly to the S2′ site (Masuyer et al., 2014). This phenomenon may explain the tiny differences in Ki between FI and FII (Masuyer et al., 2014).

#### 3.5.4 AD011, AD012, AD013

AD011, AD012, and AD013 are small molecule ACE-C/NEP dual target inhibitors recently synthesized by researchers. The crystal structure of ACE and AD011,AD012, and AD013 is depicted in Figures 5E–G. These three inhibitors are analogues of Lis-W (lisinopril derivative). Similar to Lis-W, AD011, AD012, and AD013 have higher affinity for the ACE-C domain than the ACE-N domain. Because these three inhibitors have the same backbone and P1 group and the active sites of ACE-C and ACE-N are highly similar, the interaction between these three inhibitors and the ACE domain is highly similar (Bersanetti et al., 2012). Conservative interactions in all structures include the coordination between amino acid residues (H361, H365, and E389 of ACE-N; ACE-C—H383, H387, and E411 of ACE-C) and zinc ion, as well as the coordination between the carboxyl groups in P1 group and zinc ion (Arendse et al., 2022). This carboxyl group also forms hydrogen bonds directly with E362/E384 (ACE-N/ACE-C) and Y601/Y623 (ACE-N/ACE-C) and forms water-mediated interactions with A334/A356 (ACE-N/ACE-C) and E362/E384 (ACE-N/ACE-C) (Arendse et al., 2022). The phenyl propyl group in the P1 group of the inhibitor forms a hydrophobic interaction with H331/H353 (ACE-N/ACE-C), S333/S355 (ACE-N/ACE-C), F490/F512 (ACE-N/ACE-C), and T496/V518 (ACE-N/ACE-C). Inhibitor P1′ main chain amine interact with H331/H353 and A332/A354 (ACE-N/ACE-C), while inhibitor P1′ main chain carbonyl oxygen interacts with H331/H353 and H491/H513 (ACE-N/ACE-C) (Arendse et al., 2022). The P2′ carboxylate group of the inhibitor forms a direct hydrogen bond/salt bridge with K489/K511 (ACE-N/ACE-C), Y498/Y520 (ACE-N/ACE-C) and the side chains of Q259/Q281 (ACE-N/ACE-C) and forms a water-mediated interaction with the K489/K511 (ACE-N/ACE-C) (Cozier et al., 2018b). P2′ backbone carbon atoms form hydrophobic interaction with H491/H513 (ACE-N/ACE-C) which can be found in the three inhibitors, while the hydrophobic interaction between P2′ backbone carbon atom and Y501/Y523 (ACE-N/ACE-C) is only observed in AD011 and AD012. To date, co-crystallization studies of AD011, AD012, and AD013 with NEP have not been successful. Therefore, predicting the interaction between NEP and inhibitors needs to rely on docking studies. The researchers have gained a new understanding of the factors influencing C-selectivity and NEP inhibition by synthesizing and studying the three small molecule inhibitors mentioned above, which may help to further develop a better-performing dual ACE-C/NEP inhibitor.

### 3.6 ACE inhibitors from natural resources

#### 3.6.1 Classifications of ACE inhibitors from natural resources

Because chemosynthetic ACE inhibitors have some side effects, researchers have tried to prepare ACE inhibitory substances to replace the currently available ACE inhibitors. There are four main natural sources of ACE inhibitors: plants, marine organisms, animals, and microorganisms (Table 2).

**TABLE 2:** ACE inhibitors from natural sources.

Source	Material	Substance	Structure	ACE inhibition	Reference
plant	Cecropia glazioviiSnethl	Procyanidin B2	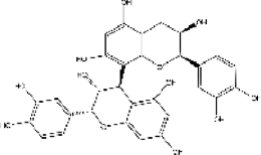	25% at 330 μg/ml	Rivera-Mondragón et al. (2017)
plant	C. glazioviiSnethl	Procyanidin C1	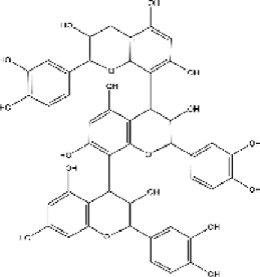	45% at 330 μg/ml	Rivera-Mondragón et al. (2017)
plant	Steviasp	Steviol glycoside	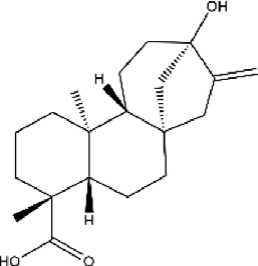	59.56% at 2500 μg/ml	Wang and Wu (2019)
plant	Astragalus membranaceus (Fisch.) Bunge	LVPPHA		IC50 = 414.88 μM	Wu et al. (2020)
plant	Limonium michelsonii	Naringenin	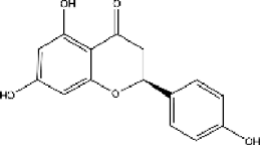	IC50 = 83.6 μM	Jenis et al. (2017)
plant	L. michelsonii	Eriodictyol	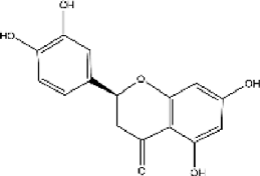	IC50 = 62.3 μM	Jenis et al. (2017)
plant	L. michelsonii	Ampelopsin	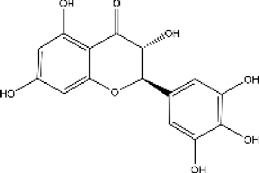	IC50 = 114.8 μM	Jenis et al. (2017)
plant	L. michelsonii	Taxifolin	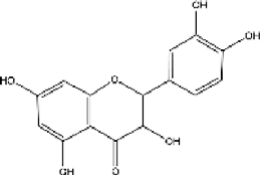	IC50 = 138.4 μM	Jenis et al. (2017)
plant	L. michelsonii	Apigenin	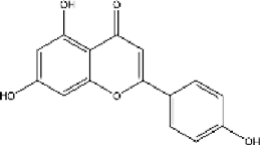	IC50 = 21.2 μM	Jenis et al. (2017)
plant	L. michelsonii	Luteolin	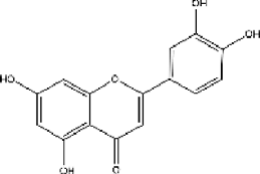	IC50 = 7.1 μM	Jenis et al. (2017)
plant	L. michelsonii	Kaempferol	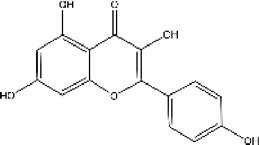	IC50 = 35.3 μM	Jenis et al. (2017)
plant	L. michelsonii	Myricetin	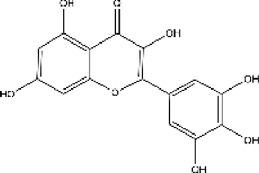	IC50 = 40.9 μM	Jenis et al. (2017)
plant	L. michelsonii	Apigenin-7-O-b-D-glucopyranoside	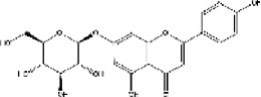	IC50 = 37.5 μM	Jenis et al. (2017)
plant	L. michelsonii	Apigenin-7-O-b-D-glucuronide	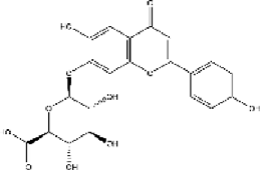	IC50 = 27.6 μM	Jenis et al. (2017)
plant	L. michelsonii	Apigenin-7-O-b-D-(600-methylglucuronide)	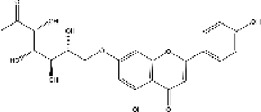	IC50 = 54.7 μM	Jenis et al. (2017)
plant	L. michelsonii	Quercetin-3-O-b-D-galactopyranoside	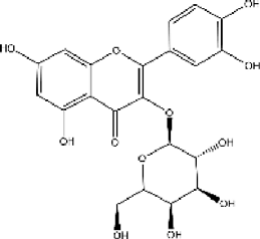	IC50 = 10.2 μM	Jenis et al. (2017)
plant	L. michelsonii	Quercetin-3-O-a-L-arabinofuranoside	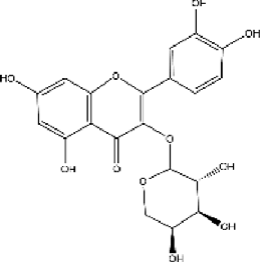	IC50 = 14.8 μM	Jenis et al. (2017)
plant	L. michelsonii	Myricetin-3-O-b-D-galactopyranoside	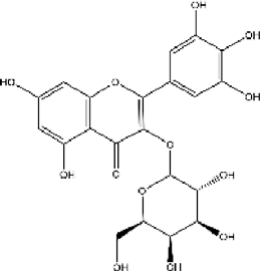	IC50 = 20.3 μM	Jenis et al. (2017)
plant	L. michelsonii	Myricetin-3-O-a-L-arabinofuranoside	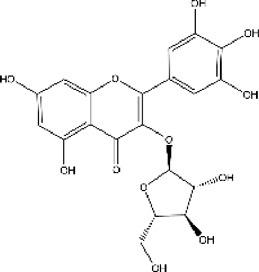	IC50 = 23.1 μM	Jenis et al. (2017)
plant	L. michelsonii	Myricetin-3-O-(600-O-galloyl)-b-D-glucopyranoside	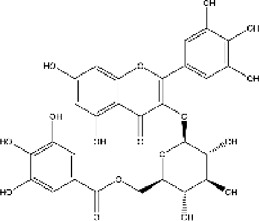	IC50 = 14.9 μM	Jenis et al. (2017)
plant	S. garrettiana	Betulinic acid	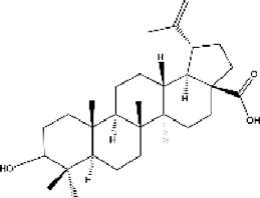	IC50 = 26.78 μM	Madaka and Charoonratana (2018)
plant	Eucommia ulmoidesOliv	Eucomegastigside A	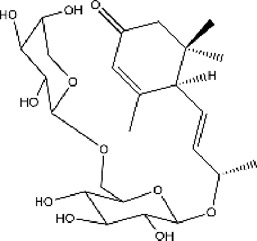	24.6% at 240 μg/ml	Yan et al. (2017)
plant	E. ulmoidesOliv	Eucomegastigside B	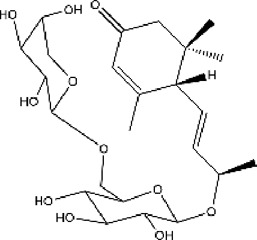	29.1% at 240 μg/ml	Yan et al. (2017)
plant	E. ulmoidesOliv	Eucomegastigside C	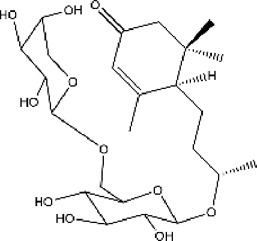	29.7% at 240 μg/ml	Yan et al. (2017)
plant	E. ulmoidesOliv	Eucomegastigside D	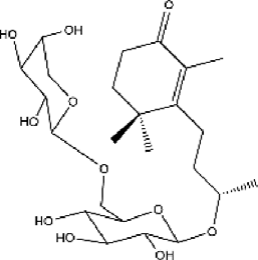	31.2% at 241 μg/ml	Yan et al. (2017)
marine organisms	Lizard fish	MKCAF		IC50 = 45.7 μM	Lan et al. (2015)
marine organisms	Lizard fish	RVCLP		IC50 = 175 μM	Wu et al. (2015)
marine organisms	Atlantic Salmon	AP		IC50 = 356.9 μM	Gu et al. (2011)
marine organisms	Atlantic Salmon	VR		IC50 = 1,301.1 μM	Gu et al. (2011)
marine organisms	Skipjack	DLDLRKDLYAN		IC50 = 67.4 μM	Intarasirisawat et al. (2013)
marine organisms	Skipjack	MCYPAST		IC50 = 58.7 μM	Intarasirisawat et al. (2013)
marine organisms	Skipjack	MLVFAV		IC50 = 3.07 μM	Intarasirisawat et al. (2013)
marine organisms	Pacific cod	GASSGMPG		IC50 = 6.9 μM	Daskaya-Dikmen et al. (2017)
marine organisms	Pacific cod	LAYA		IC50 = 14.5 μM	Daskaya-Dikmen et al. (2017)
marine organisms	Pepsin	MEVFVP		IC50 = 79 μM	Ko et al. (2016)
marine organisms	Pepsin	VSQLTR		IC50 = 105 μM	Ko et al. (2016)
marine organisms	Thermolysin	VPAAPPK		IC50 = 0.45 μM	Ghassem et al. (2011)
marine organisms	Thermolysin	NGTWFEPP		IC50 = 0.63 μM	Ghassem et al. (2011)
marine organisms	Chlorella ellipsoidea	VEGY		IC50 = 128.4 μM	Ko et al. (2012)
marine organisms	Cuttlefish	GIHETTY		IC50 = 25.66 μM	Balti et al. (2015)
marine organisms	Cuttlefish	EKSYELP		IC50 = 14.41 μM	Balti et al. (2015)
marine organisms	Cuttlefish	VELYP		IC50 = 5.22 μM	Balti et al. (2015)
marine organisms	Squid skin collagen	GRGSVPAPGP		IC50 = 47.78 μM	Alemán et al. (2013)
animal	Struthio camelus egg	WESLSRLLG		IC50 = 44.01 μM	Asoodeh et al. (2016)
animal	Egg White	TNGIIR		IC50 = 70 μM	Ding et al. (2016)
animal	Egg White	LKYAT		IC50 = 0.09 μM	Fan et al. (2019)
animal	Whey protein	LF		IC50 = 1600 μM	Guo et al. (2019)
animal	Whey protein	VFK		IC50 = 1760 μM	Guo et al. (2019)
animal	Goat milk	QSLVYPFTGPI		IC50 = 4.27 μM	Ibrahim et al. (2017)
animal	Caesin	KFPQY		IC50 = 12.37 μM	Lin et al. (2018)
animal	Caesin	NMAINPSKENLCSTFCK		IC50 = 129.07 μM	Tu et al. (2018)
animal	Bovine collagen	GPRGF		IC50 = 200.91 μM	Fu et al. (2016)

#### 3.6.2 ACE inhibitors from plants

In recent years, researchers have found ACE inhibitory plants (Chakraborty and Roy, 2021). Among their biometabolites and extracts, peptides, phenolic compounds, and terpenoids are found to possess ACE inhibitory ability. The mechanism of peptides interacting with ACE resembles those of the currently available ACEI (Jao et al., 2012). Most phenolic compounds inhibit ACE by ionic interactions with divalent zinc ions and hydrogen bonds with residues (Andrade et al., 2017). Additionally, phenolic acids such as o-carboxyl cinnamic acids inhibit ACE mainly depending on the hydrophobic effect of the benzyne ring (Al Shukor et al., 2013). Terpenoids are found to highly inhibit ACE, but their mechanism is uncertain (Andrade et al., 2017).

#### 3.6.3 ACE inhibitors from marine organisms

In recent years, researchers have found ACE inhibitory compounds from marine organisms (Pujiastuti et al., 2019). ACE inhibitory compounds inhibit ACE mainly through three modes: competitive, noncompetitive, and mixed VSQLTR from flounder fish, as a noncompetitive inhibitor, inhibits ACE by binding with the nonactive site of ACE without competition with the substrate (Ko et al., 2016). Mixed-type ACE inhibitor binding with both the active and nonactive sites leads to a change in ACE conformation and a decrease in ACE activity. Additionally, although the inhibitory mechanisms of some ACE inhibitors have been elucidated, their modes of inhibiting ACE are uncertain (Jao et al., 2012).

#### 3.6.4 ACE inhibitors from animals and animal products

In recent years, researchers have identified some ACE inhibitory compounds from animals and animal products (Wu Q. et al., 2021). Eggs and milk, which are rich in protein, are important sources for ACE processing inhibitors. Trp-Glu-Ser-Leu-Ser-Arg-Leu-Leu-Gly (WESRLLG) was extracted through hydrolysis by pepsin and pancreatin from camel eggs (Asoodeh et al., 2016).

## 4 Conclusion and perspective

### 4.1 Structure of ACE inhibitors

Today’s ACE inhibitors are mostly short peptide mimics. ACE inhibitors are typically composed of four parts: P1 group, P1′ group, P2 group, and P2′ group, which are bound to the ACE binding pockets S1, S1′, S2, and S2′, respectively through hydrogen bonds. ACE inhibitors function by sulfhydryl group/carboxylate/phosphate and divalent zinc ions. By analyzing and summarizing the structure and inhibition potency relationship of the above ACE inhibitors, we can draw the following conclusions: 1) For P1′ groups, positively charged amino acids such as lysine and arginine are preferred. 2) For P2′ groups, aromatic amino acids and proline and their derivatives are preferred, the larger the side chain, the higher the inhibition effect of ACE. 3) For the P1 group, amino acid residues need side chains containing many hydrophobic groups. By analyzing and summarizing the structure and selectivity of the above ACE inhibitors, we can draw the following conclusions: 1) Large P2′ groups contribute to ACE-C selectivity. 2) The non-conserved residues E403 and F391 in ACE-C and R381 and Y369 in ACE-N may be key locations to improve domain selectivity. In addition, the field of S3 binding pockets and the area extended by the catalytic zinc ion and core catalytic region are worthy of the attention of researchers.

### 4.2 Domain-selective ACE inhibitors and drug design

The C-domain and the N-domain are two homologous catalytic domains found in somatic ACE (Martin and Deussen, 2019). Recent research has revealed that while the two domains are architecturally quite similar. The C-domain is primarily engaged in blood pressure management, whereas the N-domain is important in hematopoietic stem cell proliferation regulation (Masuyer et al., 2012). The nonselective inhibition of these two domains in currently available ACE inhibitors results in certain negative effects. High selectivity N-domain inhibitors have also been identified as possible anti-fibrosis medicines. Current ACE medications block both areas of the enzyme, which might result in frequent adverse effects (Polakovičová and Jampílek, 2019). Developing next-generation ACEI that preferentially target ACE-C or ACE-N remains a key therapeutic aim. Selective inhibition of ACE-C lowers the negative effects of increased bradykinin and substance P, while high specific ACE-N inhibitors may be used to treat inflammation and fibrosis without the negative effects of changing blood pressure (Polakovičová and Jampílek, 2019). In the multitarget design of cardiovascular medicine, a single domain-specific ACE inhibitor should be a superior choice. The high-resolution crystal structures of ACEI with ACE-C and ACE-N may aid medication development by allowing reasonable structures to be used. The inhibitor’s selectivity for the ACE-C and ACE-N is mediated by distinct changes in distant amino acids between the S2 and S2′ subsites of the catalytic core, according to the X-ray complex (Polakovičová and Jampílek, 2019). Subtle variations in the amino acids that make up the catalytic sites govern the selectivity of ACEI for ACE-C and ACE-N. Since ACE has an effect on the cardiovascular system and also other systems, it still remains an important target where we develop new domain-selective drugs (Galanis et al., 2004).

### 4.3 ACE inhibitors from natural sources

Because existing synthetic ACEIs may lead to adverse effects such as cough, angioedema, bronchospasm, and dyskinesia, we need to develop natural, nontoxic alternatives (Zhang et al., 2022). Natural peptides from animals, plants, and marine life have recently sparked renewed attention, thanks to advances in peptide assimilation theory and biological findings. ACEIPs are the most researched natural peptides among the numerous bioactive peptides because they are safer than synthetic antihypertensive medications (Wang et al., 2020). The tissue potency is influenced by the structural and pharmacokinetic features of the medication influence (Wang et al., 2020). These natural ACEIPs offer superior nutritional value and affinity with tissues than recognized synthetic antihypertensive medicines, and are easily absorbed as a result of their amino acid makeup and sequence (Wang et al., 2020). In addition, the side effects of the ACEI mentioned above are not found in foodborne polypeptides, probably because of low blood concentrations, which are unlikely to cause common side effects. Therefore, it is necessary to accelerate the development and industrial application of foodborne angiotensin-converting enzyme inhibitors to replace existing antihypertensive drugs. There are many ways to prepare natural angiotensin-converting enzymes. Selective enzymatic methods for the preparation of peptides under mild conditions have the benefits of low cost and strong controllability and have been applied to the earlier production of crude ACEIPs (Wu et al., 2022). The isolation and purification of ACEIPs from raw materials or enzymatic digests can also be achieved by ultrafiltration membranes, gel filtration chromatography, ion exchange column chromatography and, based on known amino acid sequences, solid-phase synthesis is the preferred option to achieve large-scale preparation of ACEIPs (Wang et al., 2020). In addition to this, microbial fermentation is used as a starting culture to produce bioactive peptides *via* the non-ribosomal synthase (NRPS) pathway or by degrading endogenous microbial proteases (Wang et al., 2020). It is worthy to be noted that the synthesis of various biologically active small molecule cyclic peptides or linear peptides is mainly achieved through NRPS. However, the biosynthesis or regulatory mechanisms that produce ACEIPs in microbial cells *via* the NRPS pathway have not been studied, thereby greatly limiting the oversynthesis of ACEIPs and the production of ACEIPs through metabolic regulation. To tackle the technological barrier of ACIP oversynthesis in large-scale manufacturing, more study is needed into NRPS gene clusters, including examining the function of NRPS gene clusters in ACEIP synthesis and clarifying the mechanism (Wang et al., 2020).

## References

[B1] Acharya K. R. Sturrock E. D. Riordan J. F. Ehlers M. R. W. (2003). Ace revisited: A new target for structure-based drug design. Nat. Rev. Drug Discov. 2, 891–902. 10.1038/nrd1227 14668810 PMC7097707

[B2] Akif M. Georgiadis D. Mahajan A. Dive V. Sturrock E. D. Isaac R. E. (2010). High-resolution crystal structures of *Drosophila melanogaster* angiotensin-converting enzyme in complex with novel inhibitors and antihypertensive drugs. J. Mol. Biol. 400, 502–517. 10.1016/j.jmb.2010.05.024 20488190

[B3] Akif M. Masuyer G. Bingham R. J. Sturrock E. D. Isaac R. E. Acharya K. R. (2012). Structural basis of peptide recognition by the angiotensin-1 converting enzyme homologue AnCE from *Drosophila melanogaster* . FEBS J. 279, 4525–4534. 10.1111/febs.12038 23082758 PMC3564407

[B4] Akif M. Schwager S. L. Anthony C. S. Czarny B. Beau F. Dive V. (2011). Novel mechanism of inhibition of human angiotensin-I-converting enzyme (ACE) by a highly specific phosphinic tripeptide. Biochem. J. 436, 53–59. 10.1042/BJ20102123 21352096 PMC3086271

[B5] Al Shukor N. Van Camp J. Gonzales G. B. Staljanssens D. Struijs K. Zotti M. J. (2013). Angiotensin-converting enzyme inhibitory effects by plant phenolic compounds: A study of structure activity relationships. J. Agric. Food Chem. 61, 11832–11839. 10.1021/jf404641v 24219111

[B6] Alderman C. P. (1996). Adverse effects of the angiotensin-converting enzyme inhibitors. Ann. Pharmacother. 30, 55–61. 10.1177/106002809603000110 8773167

[B7] Alemán A. Gómez-Guillén M. C. Montero P. (2013). Identification of ace-inhibitory peptides from squid skin collagen after *in vitro* gastrointestinal digestion. Food Res. Int. 54, 790–795. 10.1016/j.foodres.2013.08.027

[B8] Alhasani K. F. Kazi M. Abbas M. Shahba A. A. Alanazi F. K. (2019). Self-nanoemulsifying ramipril tablets: A novel delivery system for the enhancement of drug dissolution and stability. Int. J. Nanomedicine 14, 5435–5448. 10.2147/IJN.S203311 31409997 PMC6645612

[B9] Andrade P. B. Valentão P. Pereira D. M. (2017). Natural products targeting clinically relevant enzymes. Hoboken, New Jersey, United States: John Wiley & Sons.

[B10] Arashi H. Sato T. Kobashigawa J. Luikart H. Kobayashi Y. Okada K. (2020). Long-term clinical outcomes with use of an angiotensin-converting enzyme inhibitor early after heart transplantation. Am. Heart J. 222, 30–37. 10.1016/j.ahj.2020.01.003 32007823

[B11] Arendse L. B. Cozier G. E. Eyermann C. J. Basarab G. S. Schwager S. L. Chibale K. (2022). Probing the requirements for dual angiotensin-converting enzyme C-domain selective/neprilysin inhibition. J. Med. Chem. 65, 3371–3387. 10.1021/acs.jmedchem.1c01924 35113565

[B12] Asoodeh A. Homayouni-Tabrizi M. Shabestarian H. Emtenani S. Emtenani S. (2016). Biochemical characterization of a novel antioxidant and angiotensin I-converting enzyme inhibitory peptide from *Struthio camelus* egg white protein hydrolysis. J. Food Drug Anal. 24, 332–342. 10.1016/j.jfda.2015.11.010 28911587 PMC9339567

[B13] Balti R. Bougatef A. Sila A. Guillochon D. Dhulster P. Nedjar-Arroume N. (2015). Nine novel angiotensin I-converting enzyme (ACE) inhibitory peptides from cuttlefish (Sepia officinalis) muscle protein hydrolysates and antihypertensive effect of the potent active peptide in spontaneously hypertensive rats. Food Chem. 170, 519–525. 10.1016/j.foodchem.2013.03.091 25306378

[B14] Barnett A. H. (2005). Preventing renal complications in diabetic patients: The diabetics exposed to telmisartan and enalaprIL (DETAIL) study. Acta Diabetol. 42, s42–s49. 10.1007/s00592-005-0180-4 15868119

[B15] Barutta F. Bellini S. Mastrocola R. Gambino R. Piscitelli F. di Marzo V. (2018). Reversal of albuminuria by combined AM6545 and perindopril therapy in experimental diabetic nephropathy: AM6545 and perindopril reverse albuminuria in diabetic mice. Br. J. Pharmacol. 175, 4371–4385. 10.1111/bph.14495 30184259 PMC6240130

[B16] Bernstein K. E. Ong F. S. Blackwell W.-L. B. Shah K. H. Giani J. F. Gonzalez-Villalobos R. A. (2013). A modern understanding of the traditional and nontraditional biological functions of angiotensin-converting enzyme. Pharmacol. Rev. 65, 1–46. 10.1124/pr.112.006809 23257181 PMC3565918

[B17] Bersanetti P. A. Sabatini R. A. Matos B. S. Douglas R. G. Nchinda A. Juliano M. A. (2012). Characterization of angiotensin I-converting enzyme N-domain selectivity using positional-scanning combinatorial libraries of fluorescence resonance energy transfer peptides. Biol. Chem. 393, 1547–1554. 10.1515/hsz-2012-0170 23667908

[B18] Biswas M. Sawajan N. Rungrotmongkol T. Sanachai K. Ershadian M. Sukasem C. (2022). Pharmacogenetics and precision medicine approaches for the improvement of COVID-19 therapies. Front. Pharmacol. 13, 835136. 10.3389/fphar.2022.835136 35250581 PMC8894812

[B19] Borchert T. Hess A. Lukačević M. Ross T. L. Bengel F. M. Thackeray J. T. (2020). Angiotensin-converting enzyme inhibitor treatment early after myocardial infarction attenuates acute cardiac and neuroinflammation without effect on chronic neuroinflammation. Eur. J. Nucl. Med. Mol. Imaging 47, 1757–1768. 10.1007/s00259-020-04736-8 32125488 PMC7248052

[B20] Bullo M. Tschumi S. Bucher B. S. Bianchetti M. G. Simonetti G. D. (2012). Pregnancy outcome following exposure to angiotensin-converting enzyme inhibitors or angiotensin receptor antagonists: A systematic review. Hypertension 60, 444–450. 10.1161/HYPERTENSIONAHA.112.196352 22753220

[B21] Caletti M. G. Lejarraga H. Kelmansky D. Missoni M. (2004). Two different therapeutic regimes in patients with sequelae of hemolytic-uremic syndrome. Pediatr. Nephrol. 19, 1148–1152. 10.1007/s00467-004-1516-y 15221428

[B22] Campbell D. J. Zeitz C. J. Esler M. D. Horowitz J. D. (2004). Evidence against a major role for angiotensin converting enzyme-related carboxypeptidase (ACE2) in angiotensin peptide metabolism in the human coronary circulation. J. Hypertens. 22, 1971–1976. 10.1097/00004872-200410000-00020 15361769

[B23] Chakraborty R. Roy S. (2021). Angiotensin-converting enzyme inhibitors from plants: A review of their diversity, modes of action, prospects, and concerns in the management of diabetes-centric complications. J. Integr. Med. 19, 478–492. 10.1016/j.joim.2021.09.006 34642085

[B24] Chauhan V. P. Martin J. D. Liu H. Lacorre D. A. Jain S. R. Kozin S. V. (2013). Angiotensin inhibition enhances drug delivery and potentiates chemotherapy by decompressing tumour blood vessels. Nat. Commun. 4, 2516. 10.1038/ncomms3516 24084631 PMC3806395

[B25] Chen A. Y. Adamek R. N. Dick B. L. Credille C. V. Morrison C. N. Cohen S. M. (2019). Targeting metalloenzymes for therapeutic intervention. Chem. Rev. 119, 1323–1455. 10.1021/acs.chemrev.8b00201 30192523 PMC6405328

[B26] Corradi H. R. Chitapi I. Sewell B. T. Georgiadis D. Dive V. Sturrock E. D. (2007). The structure of testis angiotensin-converting enzyme in complex with the C domain-specific inhibitor RXPA380. Biochemistry 46, 5473–5478. 10.1021/bi700275e 17439247

[B27] Corradi H. R. Schwager S. L. U. Nchinda A. T. Sturrock E. D. Acharya K. R. (2006). Crystal structure of the N domain of human somatic angiotensin I-converting enzyme provides a structural basis for domain-specific inhibitor design. J. Mol. Biol. 357, 964–974. 10.1016/j.jmb.2006.01.048 16476442

[B28] Cozier G. E. Arendse L. B. Schwager S. L. Sturrock E. D. Acharya K. R. (2018a). Molecular basis for multiple omapatrilat binding sites within the ACE C-domain: Implications for drug design. J. Med. Chem. 61, 10141–10154. 10.1021/acs.jmedchem.8b01309 30372620

[B29] Cozier G. E. Newby E. C. Schwager S. L. U. Isaac R. E. Sturrock E. D. Acharya K. R. (2022). Structural basis for the inhibition of human angiotensin‐1 converting enzyme by fosinoprilat. FEBS J., 16543. 10.1111/febs.16543 PMC9796954 35653492

[B30] Cozier G. E. Schwager S. L. Sharma R. K. Chibale K. Sturrock E. D. Acharya K. R. (2018b). Crystal structures of sampatrilat and sampatrilat-asp in complex with human ACE – A molecular basis for domain selectivity. FEBS J. 285, 1477–1490. 10.1111/febs.14421 29476645 PMC5947662

[B31] D’Agati V. D. (2017). Podocyte growing pains in adaptive FSGS. J. Am. Soc. Nephrol. 28, 2825–2827. 10.1681/ASN.2017060612 28716959 PMC5619978

[B32] Daskaya-Dikmen C. Yucetepe A. Karbancioglu-Guler F. Daskaya H. Ozcelik B. (2017). Angiotensin-I-Converting enzyme (ACE)-Inhibitory peptides from plants. Nutrients 9, E316. 10.3390/nu9040316 28333109 PMC5409655

[B33] Dicpinigaitis P. V. (2006). Angiotensin-converting enzyme inhibitor-induced cough: ACCP evidence-based clinical practice guidelines. Chest 129, 169S–173S. 10.1378/chest.129.1_suppl.169S 16428706

[B34] Ding L. Wang L. Yu Z. Zhang T. Liu J. (2016). Digestion and absorption of an egg white ACE-inhibitory peptide in human intestinal Caco-2 cell monolayers. Int. J. Food Sci. Nutr. 67, 111–116. 10.3109/09637486.2016.1144722 26883099

[B35] Douros A. Kauffmann W. Bronder E. Klimpel A. Garbe E. Kreutz R. (2013). Ramipril-Induced liver injury: Case report and review of the literature. Am. J. Hypertens. 26, 1070–1075. 10.1093/ajh/hpt090 23747952

[B36] Duvančić T. Lugović-Mihić L. Brekalo A. Šitum M. Šinković A. (2011). Prominent features of allergic angioedema on oral mucosa. Acta Clin. Croat. 50, 531–538. 22649883

[B37] Egan B. M. (2007). Combination therapy with an angiotensin-converting enzyme inhibitor and a calcium channel blocker. J. Clin. Hypertens. 9, 783–789. 10.1111/j.1751-7176.2007.tb00005.x PMC8110094 17917506

[B38] Ehlers M. R. W. Riordan J. F. (2002). Angiotensin-converting enzyme: Zinc- and inhibitor-binding stoichiometries of the somatic and testis isozymes. Washington D. C.: ACS Publications. 10.1021/bi00243a012 1649623

[B39] Engineer D. R. Burney B. O. Hayes T. G. Garcia J. M. (2013). Exposure to ACEI/ARB and β-blockers is associated with improved survival and decreased tumor progression and hospitalizations in patients with advanced colon cancer. Transl. Oncol. 6, 539–545. 10.1593/tlo.13346 24151534 PMC3799196

[B40] Esposito R. Giammarino A. De Blasio A. Martinelli V. Cirillo F. Scopacasa F. (2007). Ramipril in post-renal transplant erythrocytosis. J. Nephrol. 20, 57–62. Available at: https://pubmed.ncbi.nlm.nih.gov/17347974/Accessed December 25, 2021). 17347974

[B41] Evans M. Carrero J.-J. Szummer K. Åkerblom A. Edfors R. Spaak J. (2016). Angiotensin-converting enzyme inhibitors and angiotensin receptor blockers in myocardial infarction patients with renal dysfunction. J. Am. Coll. Cardiol. 67, 1687–1697. 10.1016/j.jacc.2016.01.050 27056774

[B42] Fan H. F. Xun Z. Hua Z. G. Di X. Yan C. P. Ru Z. W. (2006). Efficacy and safety of benazepril for advanced chronic renal insufficiency. N. Engl. J. Med. 10, 131–140. 10.1056/NEJMoa053107 16407508

[B43] Fan H. Wang J. Liao W. Jiang X. Wu J. (2019). Identification and characterization of gastrointestinal-resistant angiotensin-converting enzyme inhibitory peptides from egg white proteins. J. Agric. Food Chem. 67, 7147–7156. 10.1021/acs.jafc.9b01071 31140270

[B44] Fearon W. F. Okada K. Kobashigawa J. A. Kobayashi Y. Luikart H. Sana S. (2017). Angiotensin-converting enzyme inhibition early after heart transplantation. J. Am. Coll. Cardiol. 69, 2832–2841. 10.1016/j.jacc.2017.03.598 28595700 PMC5546225

[B45] Fernandez M. Liu X. Wouters M. A. Heyberger S. Husain A. (2001). Angiotensin I-converting enzyme transition state stabilization by His1089: Evidence for a catalytic mechanism distinct from other gluzincin metalloproteinases. J. Biol. Chem. 276, 4998–5004. 10.1074/jbc.M009009200 11067854

[B46] Fienberg S. Cozier G. E. Acharya K. R. Chibale K. Sturrock E. D. (2018). The design and development of a potent and selective novel diprolyl derivative that binds to the N-domain of angiotensin-I converting enzyme. J. Med. Chem. 61, 344–359. 10.1021/acs.jmedchem.7b01478 29206036

[B47] Firouzabadi N. Farshadfar P. Haghnegahdar M. Alavi-Shoushtari A. Ghanbarinejad V. (2022). Impact of ACE2 genetic variant on antidepressant efficacy of SSRIs. Acta Neuropsychiatr. 34, 30–36. 10.1017/neu.2021.32 34602110

[B48] Fischer N. M. Nieuwenhuis T. O. Singh B. Yenokyan G. Segars J. H. (2021). Angiotensin-converting enzyme inhibitors reduce uterine fibroid incidence in hypertensive women. J. Clin. Endocrinol. Metab. 106, e650–e659. 10.1210/clinem/dgaa718 33035320 PMC7823233

[B49] Frohlich E. D. Cooper R. A. Lewis E. J. (1984). Review of the overall experience of captopril in hypertension. Arch. Intern. Med. 144, 1441–1444. 10.1001/archinte.144.7.1441 6233948

[B50] Fu Y. Young J. F. Rasmussen M. K. Dalsgaard T. K. Lametsch R. Aluko R. E. (2016). Angiotensin I–converting enzyme–inhibitory peptides from bovine collagen: Insights into inhibitory mechanism and transepithelial transport. Food Res. Int. 89, 373–381. 10.1016/j.foodres.2016.08.037 28460927

[B51] Fuchs S. Xiao H. D. Hubert C. Michaud A. Campbell D. J. Adams J. W. (2008). Angiotensin-converting enzyme C-terminal catalytic domain is the main site of angiotensin I cleavage *in vivo* . Hypertension 51, 267–274. 10.1161/HYPERTENSIONAHA.107.097865 18158355

[B52] Fukuda A. Chowdhury M. A. Venkatareddy M. P. Wang S. Q. Nishizono R. Suzuki T. (2012). Growth-dependent podocyte failure causes glomerulosclerosis. J. Am. Soc. Nephrol. 23, 1351–1363. 10.1681/ASN.2012030271 22773827 PMC3402293

[B53] Fukuda D. Sata M. (2008). Role of bone marrow renin-angiotensin system in the pathogenesis of atherosclerosis. Pharmacol. Ther. 118, 268–276. 10.1016/j.pharmthera.2008.02.007 18439685

[B54] Galanis A. S. Spyroulias G. A. Pairas G. Manessi-Zoupa E. Cordopatis P. (2004). Solid-phase synthesis and conformational properties of angiotensin converting enzyme catalytic-site peptides: The basis for a structural study on the enzyme-substrate interaction. Biopolymers 76, 512–526. 10.1002/bip.20163 15508121

[B55] Ghassem M. Arihara K. Babji A. S. Said M. Ibrahim S. (2011). Purification and identification of ACE inhibitory peptides from Haruan (Channa striatus) myofibrillar protein hydrolysate using HPLC–ESI-TOF MS/MS. Food Chem. 129, 1770–1777. 10.1016/j.foodchem.2011.06.051

[B56] Gu R.-Z. Li C.-Y. Liu W.-Y. Yi W.-X. Cai M.-Y. (2011). Angiotensin I-converting enzyme inhibitory activity of low-molecular-weight peptides from Atlantic salmon (*Salmo salar* L.) skin. Food Res. Int. 44, 1536–1540. 10.1016/j.foodres.2011.04.006

[B57] Guo Y. Jiang X. Xiong B. Zhang T. Zeng X. Wu Z. (2019). Production and transepithelial transportation of angiotensin-I-converting enzyme (ACE)-inhibitory peptides from whey protein hydrolyzed by immobilized Lactobacillus helveticus proteinase. J. Dairy Sci. 102, 961–975. 10.3168/jds.2018-14899 30594363

[B58] Hanif K. Bid H. K. Konwar R. (2010). Reinventing the ACE inhibitors: Some old and new implications of ACE inhibition. Hypertens. Res. 33, 11–21. 10.1038/hr.2009.184 19911001

[B59] Harahap Y. Prasetyo V. Sandra M. Rahayu T. Lusthom W. Prasaja B. (2021). Comparative bioavailability study of two imidapril tablet formulations in Indonesian healthy subjects. Syst. Rev. Pharm. 12, 5.

[B60] Heran B. S. Wong M. M. Heran I. K. Wright J. M. (2008). Blood pressure lowering efficacy of angiotensin converting enzyme (ACE) inhibitors for primary hypertension. Cochrane Database Syst. Rev. 2008, CD003823. 10.1002/14651858.CD003823.pub2 PMC7156914 18843651

[B61] Herman L. L. Padala S. A. Ahmed I. Bashir K. (2021). Angiotensin converting enzyme inhibitors (ACEI). StatPearls Publishing Available at: https://www.ncbi.nlm.nih.gov/books/NBK431051/Accessed date January 2, 2022]. 28613705

[B62] Hirayama Y. Atarashi H. Kobayashi Y. Horie T. Iwasaki Y. Maruyama M. (2005). Angiotensin-converting enzyme inhibitor therapy inhibits the progression from paroxysmal atrial fibrillation to chronic atrial fibrillation. Circ. J. 69, 671–676. 10.1253/circj.69.671 15914944

[B63] Hsieh Y.-C. Hung C.-Y. Li C.-H. Liao Y.-C. Huang J.-L. Lin C.-H. (2016). Angiotensin-receptor blocker, angiotensin-converting enzyme inhibitor, and risks of atrial fibrillation: A nationwide cohort study. Med. Baltim. 95, e3721. 10.1097/MD.0000000000003721 PMC4902433 27196491

[B64] Hui K. K. Duchin K. L. Kripalani K. J. Chan D. Kramer P. K. Yanagawa N. (1991). Pharmacokinetics of fosinopril in patients with various degrees of renal function. Clin. Pharmacol. Ther. 49, 457–467. 10.1038/clpt.1991.54 1826651

[B65] Ibrahim H. R. Ahmed A. S. Miyata T. (2017). Novel angiotensin-converting enzyme inhibitory peptides from caseins and whey proteins of goat milk. J. Adv. Res. 8, 63–71. 10.1016/j.jare.2016.12.002 28053783 PMC5196233

[B66] Intarasirisawat R. Benjakul S. Wu J. Visessanguan W. (2013). Isolation of antioxidative and ACE inhibitory peptides from protein hydrolysate of skipjack (Katsuwana pelamis) roe. J. Funct. Foods 5, 1854–1862. 10.1016/j.jff.2013.09.006

[B67] Iwaniak A. Minkiewicz P. Darewicz M. (2014). Food-originating ACE inhibitors, including antihypertensive peptides, as preventive food components in blood pressure reduction. Compr. Rev. Food Sci. Food Saf. 13, 114–134. 10.1111/1541-4337.12051 33412648

[B68] James P. A. Oparil S. Carter B. L. Cushman W. C. Dennison-Himmelfarb C. Handler J. (2014). 2014 evidence-based guideline for the management of high blood pressure in adults: Report from the panel members appointed to the eighth joint national committee (JNC 8). JAMA 311, 507–520. 10.1001/jama.2013.284427 24352797

[B69] Jao C.-L. Huang S.-L. Hsu K.-C. (2012). Angiotensin I-converting enzyme inhibitory peptides: Inhibition mode, bioavailability, and antihypertensive effects. BioMedicine 2, 130–136. 10.1016/j.biomed.2012.06.005

[B70] Jenis J. Kim J. Y. Uddin Z. Song Y. H. Lee H.-H. Park K. H. (2017). Phytochemical profile and angiotensin I converting enzyme (ACE) inhibitory activity of Limonium michelsonii Lincz. J. Nat. Med. 71, 650–658. 10.1007/s11418-017-1095-4 28550653

[B71] Kai H. Kai M. (2020). Interactions of coronaviruses with ACE2, angiotensin II, and RAS inhibitors-lessons from available evidence and insights into COVID-19. Hypertens. Res. 43, 648–654. 10.1038/s41440-020-0455-8 32341442 PMC7184165

[B72] Khurana V. Goswami B. (2022). Angiotensin converting enzyme (ACE). Clin. Chim. Acta. 524, 113–122. 10.1016/j.cca.2021.10.029 34728179

[B73] Ko J.-Y. Kang N. Lee J.-H. Kim J.-S. Kim W.-S. Park S.-J. (2016). Angiotensin I-converting enzyme inhibitory peptides from an enzymatic hydrolysate of flounder fish ( *Paralichthys olivaceus* ) muscle as a potent anti-hypertensive agent. Process Biochem. 51, 535–541. 10.1016/j.procbio.2016.01.009

[B74] Ko S.-C. Kang N. Kim E.-A. Kang M. C. Lee S.-H. Kang S.-M. (2012). A novel angiotensin I-converting enzyme (ACE) inhibitory peptide from a marine Chlorella ellipsoidea and its antihypertensive effect in spontaneously hypertensive rats. Process Biochem. 47, 2005–2011. 10.1016/j.procbio.2012.07.015

[B75] Kostis W. J. Shetty M. Chowdhury Y. S. Kostis J. B. (2018). ACE inhibitor-induced angioedema: A review. Curr. Hypertens. Rep. 20, 55. 10.1007/s11906-018-0859-x 29884969

[B76] Kramer G. J. Mohd A. Schwager S. L. U. Masuyer G. Acharya K. R. Sturrock E. D. (2014). Interkingdom pharmacology of angiotensin-I converting enzyme inhibitor phosphonates produced by actinomycetes. ACS Med. Chem. Lett. 5, 346–351. 10.1021/ml4004588 24900839 PMC4027624

[B77] Kröger W. L. Douglas R. G. O’Neill H. G. Dive V. Sturrock E. D. (2009). Investigating the domain specificity of phosphinic inhibitors RXPA380 and RXP407 in angiotensin-converting enzyme. Biochemistry 48, 8405–8412. 10.1021/bi9011226 19658433

[B78] Kumagai K. (2007). Upstream therapy for atrial fibrillation. Circ. J. 71, A75–A81. 10.1253/circj.71.a75 17587744

[B79] Kumar R. Sharma R. Bairwa K. Roy R. K. Kumar A. Baruwa A. (2010). Modern development in ACE inhibitors. Lettre, Der Pharmacia 2 (3), 388–419.

[B80] Lan X. Liao D. Wu S. Wang F. Sun J. Tong Z. (2015). Rapid purification and characterization of angiotensin converting enzyme inhibitory peptides from lizard fish protein hydrolysates with magnetic affinity separation. Food Chem. 182, 136–142. 10.1016/j.foodchem.2015.02.004 25842319

[B81] Lin H. Geurts F. Hassler L. Batlle D. Mirabito Colafella K. M. Denton K. M. (2022). Kidney angiotensin in cardiovascular disease: Formation and drug targeting. Pharmacol. Rev. 74, 462–505. 10.1124/pharmrev.120.000236 35710133 PMC9553117

[B82] Lin K. Zhang L. Han X. Meng Z. Zhang J. Wu Y. (2018). Quantitative structure–activity relationship modeling coupled with molecular docking analysis in screening of angiotensin I-converting enzyme inhibitory peptides from qula casein hydrolysates obtained by two-enzyme combination hydrolysis. J. Agric. Food Chem. 66, 3221–3228. 10.1021/acs.jafc.8b00313 29521090

[B83] Lv B. Chen S. Tang C. Jin H. Du J. Huang Y. (2021). Hydrogen sulfide and vascular regulation – an update. J. Adv. Res. 27, 85–97. 10.1016/j.jare.2020.05.007 33318869 PMC7728588

[B84] Macabrey D. Deslarzes-Dubuis C. Longchamp A. Lambelet M. Ozaki C. K. Corpataux J.-M. (2021). Hydrogen sulphide release via the angiotensin converting enzyme inhibitor zofenopril prevents intimal hyperplasia in human vein segments and in a mouse model of carotid artery stenosis. Eur. J. Vasc. Endovascular Surg. 63 (2), 336–346. S1078588421007711. 10/gnv7bk. 10.1016/j.ejvs.2021.09.032 34916111

[B85] Madaka F. Charoonratana T. (2018). Angiotensin-converting enzyme inhibitory activity of Senna garrettiana active compounds: Potential markers for standardized herbal medicines. Pharmacogn. Mag. 14, 335. 10.4103/pm.pm_325_17

[B86] Mak K. Y. Chin R. Cunningham S. C. Habib M. R. Torresi J. Sharland A. F. (2015). ACE2 therapy using adeno-associated viral vector inhibits liver fibrosis in mice. Mol. Ther. 23, 1434–1443. 10.1038/mt.2015.92 25997428 PMC4817885

[B87] Marin R. Ruilope L. M. Aljama P. Aranda P. Segura J. Diez J. (2001). A random comparison of fosinopril and nifedipine GITS in patients with primary renal disease. J. Hypertens. 19, 1871–1876. 10.1097/00004872-200110000-00023 11593109

[B88] Martin M. Deussen A. (2019). Effects of natural peptides from food proteins on angiotensin converting enzyme activity and hypertension. Crit. Rev. Food Sci. Nutr. 59, 1264–1283. 10.1080/10408398.2017.1402750 29244531

[B89] Masuyer G. Akif M. Czarny B. Beau F. Schwager S. L. U. Sturrock E. D. (2014). Crystal structures of highly specific phosphinic tripeptide enantiomers in complex with the angiotensin‐ I converting enzyme. FEBS J. 281, 943–956. 10.1111/febs.12660 24289879 PMC4154125

[B90] Masuyer G. Schwager S. L. U. Sturrock E. D. Isaac R. E. Acharya K. R. (2012). Molecular recognition and regulation of human angiotensin-I converting enzyme (ACE) activity by natural inhibitory peptides. Sci. Rep. 2, 717. 10.1038/srep00717 23056909 PMC3466449

[B91] Mohanty S. Mohanty P. Trivedi C. Gianni C. Bai R. Burkhardt J. D. (2015). Association of pretreatment with angiotensin-converting enzyme inhibitors with improvement in ablation outcome in atrial fibrillation patients with low left ventricular ejection fraction. Heart rhythm. 12, 1963–1971. 10.1016/j.hrthm.2015.06.007 26051531

[B92] Moratal C. Laurain A. Naïmi M. Florin T. Esnault V. Neels J. G. (2021). Regulation of monocytes/macrophages by the renin–angiotensin system in diabetic nephropathy: State of the art and results of a pilot study. Int. J. Mol. Sci. 22, 6009. 10.3390/ijms22116009 34199409 PMC8199594

[B93] Munavalli G. G. Knutsen-Larson S. Lupo M. P. Geronemus R. G. (2021). Oral angiotensin-converting enzyme inhibitors for treatment of delayed inflammatory reaction to dermal hyaluronic acid fillers following COVID-19 vaccination-a model for inhibition of angiotensin II–induced cutaneous inflammation. JAAD Case Rep. 10, 63–68. 10.1016/j.jdcr.2021.02.018 33681439 PMC7923909

[B94] Naik A. S. Wang S. Q. Chowdhury M. Aqeel J. O’Connor C. L. Wiggins J. E. (2021). Critical timing of ACEi initiation prevents compensatory glomerular hypertrophy in the remaining single kidney. Sci. Rep. 11, 19605. 10.1038/s41598-021-99124-z 34599260 PMC8486841

[B95] Natesh R. Schwager S. L. U. Evans H. R. Sturrock E. D. Acharya K. R. (2004). Structural details on the binding of antihypertensive drugs captopril and enalaprilat to human testicular angiotensin I-converting enzyme. Biochemistry 43, 8718–8724. 10.1021/bi049480n 15236580

[B96] Natesh R. Schwager S. L. U. Sturrock E. D. Acharya K. R. (2003). Crystal structure of the human angiotensin-converting enzyme–lisinopril complex. Nature 421, 551–554. 10.1038/nature01370 12540854

[B97] Navis G. Faber H. J. de Zeeuw D. de Jong P. E. (1996). ACE inhibitors and the kidney. A risk-benefit assessment. Drug Saf. 15, 200–211. 10.2165/00002018-199615030-00005 8879974

[B98] Neves M. F. Cunha A. R. Cunha M. R. Gismondi R. A. Oigman W. (2018). The role of renin-angiotensin-aldosterone system and its new components in arterial stiffness and vascular aging. High. Blood Press. Cardiovasc. Prev. 25, 137–145. 10.1007/s40292-018-0252-5 29476451

[B99] Nordenskjöld A. M. Agewall S. Atar D. Baron T. Beltrame J. Bergström O. (2021). Randomized evaluation of beta blocker and ACE-inhibitor/angiotensin receptor blocker treatment in patients with myocardial infarction with non-obstructive coronary arteries (MINOCA-BAT): Rationale and design. Am. Heart J. 231, 96–104. 10.1016/j.ahj.2020.10.059 33203618

[B100] O’Gara P. T. Kushner F. G. Ascheim D. D. Casey D. E. Chung M. K. de Lemos J. A. (2013). 2013 ACCF/AHA guideline for the management of ST-elevation myocardial infarction: A report of the American college of cardiology foundation/American heart association task force on practice guidelines. Circulation 127, e362–e425. 10.1161/CIR.0b013e3182742cf6 23247304

[B101] Oz M. Lorke D. E. Kabbani N. (2021). A comprehensive guide to the pharmacologic regulation of angiotensin converting enzyme 2 (ACE2), the SARS-CoV-2 entry receptor. Pharmacol. Ther. 221, 107750. 10.1016/j.pharmthera.2020.107750 33275999 PMC7854082

[B102] Ozawa T. Hashiguchi Y. Yagi T. Fukushima Y. Shimada R. Hayama T. (2019). Angiotensin I-converting enzyme inhibitors/angiotensin II receptor blockers may reduce tumor recurrence in left-sided and early colorectal cancers. Int. J. Colorectal Dis. 34, 1731–1739. 10.1007/s00384-019-03379-y 31478086

[B103] Pal Khaket T. Singh J. Attri P. Dhanda S. (2012). Enkephalin degrading enzymes: Metalloproteases with high potential for drug development. Curr. Pharm. Des. 18, 220–230. 10.2174/138161212799040547 22229560

[B104] Panday S. K. (2011). Advances in the chemistry of proline and its derivatives: An excellent amino acid with versatile applications in asymmetric synthesis. Tetrahedron Asymmetry 22, 1817–1847. 10.1016/j.tetasy.2011.09.013

[B105] Papadopoulos D. P. Economou E. V. Makris T. K. Kapetanios K. J. Moyssakis I. Votteas V. E. (2004). Effect of angiotensin-converting enzyme inhibitor on collagenolytic enzyme activity in patients with acute myocardial infarction. Drugs Exp. Clin. Res. 30, 55–65. 15272643

[B106] Papadopoulos D. P. Moyssakis I. Makris T. K. Poulakou M. Stavroulakis G. Perrea D. (2005). Clinical significance of matrix metalloproteinases activity in acute myocardial infarction. Eur. Cytokine Netw. 16, 152–160. 15941687

[B107] Perini M. V. Dmello R. S. Nero T. L. Chand A. L. (2020). Evaluating the benefits of renin-angiotensin system inhibitors as cancer treatments. Pharmacol. Ther. 211, 107527. 10.1016/j.pharmthera.2020.107527 32173557

[B108] Pinto B. Jadhav U. Singhai P. Sadhanandham S. Shah N. (2020). ACEI-induced cough: A review of current evidence and its practical implications for optimal CV risk reduction. Indian Heart J. 72, 345–350. 10.1016/j.ihj.2020.08.007 33189192 PMC7670268

[B109] Polakovičová M. Jampílek J. (2019). Advances in structural biology of ACE and development of domain selective ACE-inhibitors. Med. Chem. 15, 574–587. 10.2174/1573406415666190514081132 31084594

[B110] Ponikowski P. Voors A. A. Anker S. D. Bueno H. Cleland J. G. F. Coats A. J. S. (2016). 2016 ESC Guidelines for the diagnosis and treatment of acute and chronic heart failure: The Task Force for the diagnosis and treatment of acute and chronic heart failure of the European Society of Cardiology (ESC)Developed with the special contribution of the Heart Failure Association (HFA) of the ESC. Eur. Heart J. 37, 2129–2200. 10.1093/eurheartj/ehw128 27206819

[B111] Pujiastuti D. Y. Ghoyatul Amin M. N. Alamsjah M. A. Hsu J.-L. (2019). Marine organisms as potential sources of bioactive peptides that inhibit the activity of angiotensin I-converting enzyme: A review. Molecules 24, 2541. 10.3390/molecules24142541 PMC6680877 31336853

[B112] Redón J. Trenkwalder P. R. A. Barrios V. (2013). Efficacy of combination therapy with angiotensin-converting enzyme inhibitor and calcium channel blocker in hypertension. Expert Opin. Pharmacother. 14, 155–164. 10.1517/14656566.2013.748037 23194194

[B113] Remko M. Bojarska J. Remková A. Maniukiewicz W. (2015). Molecular structure and acidity of captopril, zofenopril and their metabolites captopril disulfide and zofenoprilat. Comput. Theor. Chem. 1062, 50–55. 10.1016/j.comptc.2015.03.025

[B114] Rico-Mesa J. S. White A. Anderson A. S. (2020). Outcomes in patients with COVID-19 infection taking ACEI/ARB. Curr. Cardiol. Rep. 22, 31. 10.1007/s11886-020-01291-4 32291526 PMC7154066

[B115] Riddiough G. E. Fifis T. Walsh K. A. Muralidharan V. Christophi C. Tran B. M. (2021). Captopril, a renin-angiotensin system inhibitor, attenuates features of tumor invasion and down-regulates C-myc expression in a mouse model of colorectal cancer liver metastasis. Cancers 13, 2734. 10.3390/cancers13112734 34073112 PMC8199217

[B116] Rivera-Mondragón A. Ortíz O. O. Bijttebier S. Vlietinck A. Apers S. Pieters L. (2017). Selection of chemical markers for the quality control of medicinal plants of the genus *Cecropia* . Pharm. Biol. 55, 1500–1512. 10.1080/13880209.2017.1307421 28372473 PMC6130728

[B117] Rosenthal T. Gavras I. (2019). Renin-angiotensin inhibition in combating malignancy: A review. Anticancer Res. 39, 4597–4602. 10.21873/anticanres.13639 31519556

[B118] Salvetti A. (1990). Newer ACE inhibitors. A look at the future. Drugs 40, 800–828. 10.2165/00003495-199040060-00004 2078997

[B119] Santos R. A. S. Ferreira A. J. Verano-Braga T. Bader M. (2013). Angiotensin-converting enzyme 2, angiotensin-(1-7) and mas: New players of the renin-angiotensin system. J. Endocrinol. 216, R1–R17. 10.1530/JOE-12-0341 23092879

[B120] Santos R. A. S. Sampaio W. O. Alzamora A. C. Motta-Santos D. Alenina N. Bader M. (2018). The ACE2/angiotensin-(1-7)/MAS Axis of the renin-angiotensin system: Focus on angiotensin-(1-7). Physiol. Rev. 98, 505–553. 10.1152/physrev.00023.2016 29351514 PMC7203574

[B121] Sharma R. K. Espinoza-Moraga M. Poblete H. Douglas R. G. Sturrock E. D. Caballero J. (2016). The dynamic nonprime binding of sampatrilat to the C-domain of angiotensin-converting enzyme. J. Chem. Inf. Model. 56, 2486–2494. 10.1021/acs.jcim.6b00524 27959521

[B122] Sharma U. Cozier G. E. Sturrock E. D. Acharya K. R. (2020). Molecular basis for omapatrilat and sampatrilat binding to neprilysin-implications for dual inhibitor design with angiotensin-converting enzyme. J. Med. Chem. 63, 5488–5500. 10.1021/acs.jmedchem.0c00441 32337993 PMC7304895

[B123] Sica D. A. Cutler R. E. Parmer R. J. Ford N. F. (1991). Comparison of the steady-state pharmacokinetics of fosinopril, lisinopril and enalapril in patients with chronic renal insufficiency. Clin. Pharmacokinet. 20, 420–427. 10.2165/00003088-199120050-00006 1652404

[B124] Singh R. Rathore S. S. Khan H. Bhurwal A. Sheraton M. Ghosh P. (2021). Mortality and severity in COVID-19 patients on ACEIs and ARBs-A systematic review, meta-analysis, and meta-regression analysis. Front. Med. 8, 703661. 10.3389/fmed.2021.703661 PMC8784609 35083229

[B125] Sinnott S.-J. Douglas I. J. Smeeth L. Williamson E. Tomlinson L. A. (2020). First line drug treatment for hypertension and reductions in blood pressure according to age and ethnicity: Cohort study in UK primary care. BMJ 371, m4080. 10.1136/bmj.m4080 33208355 PMC7670766

[B126] Skidgel R. A. (1992). Bradykinin-degrading enzymes: Structure, function, distribution, and potential roles in cardiovascular pharmacology. J. Cardiovasc. Pharmacol. 20 (9), S4–S9. 10.1097/00005344-199200209-00003 1282629

[B127] Skirgello O. E. Balyasnikova I. V. Binevski P. V. Sun Z.-L. Baskin I. I. Palyulin V. A. (2006). Inhibitory antibodies to human angiotensin-converting enzyme: Fine epitope mapping and mechanism of action. Biochemistry 45, 4831–4847. 10.1021/bi052591h 16605251

[B128] Smith J. T. (1972). Primary examiner-donald G. Daus assistant examiner-mark L. Berch attorney, agent, or firm-lawrence S. Levinson; merle J. Smith. Chem. Abs. 77, 33851w.

[B129] Sriram K. Insel P. A. (2020). Risks of ACE inhibitor and ARB usage in COVID-19: Evaluating the evidence. Clin. Pharmacol. Ther. 108, 236–241. 10.1002/cpt.1863 32320478 PMC7264499

[B130] Steinhauff S. Pehlivanli S. Bakovic-Alt R. Meiser B. M. Becker B. F. von Scheidt W. (2004). Beneficial effects of quinaprilat on coronary vasomotor function, endothelial oxidative stress, and endothelin activation after human heart transplantation. Transplantation 77, 1859–1865. 10.1097/01.tp.0000131148.78203.b7 15223904

[B131] Strippoli G. F. M. Craig M. Schena F. P. Craig J. C. (2005). Antihypertensive agents for primary prevention of diabetic nephropathy. J. Am. Soc. Nephrol. 16, 3081–3091. 10.1681/ASN.2004080634 16135776

[B132] Tabacova S. (2005). Mode of action: Angiotensin-converting enzyme inhibition--developmental effects associated with exposure to ACE inhibitors. Crit. Rev. Toxicol. 35, 747–755. 10.1080/10408440591007160 16417042

[B133] Tan N.-D. Qiu Y. Xing X.-B. Ghosh S. Chen M.-H. Mao R. (2020). Associations between angiotensin-converting enzyme inhibitors and angiotensin II receptor blocker use, gastrointestinal symptoms, and mortality among patients with COVID-19. Gastroenterology 159, 1170–1172.e1. 10.1053/j.gastro.2020.05.034 32422208 PMC7228878

[B134] Tan W.-Q. Fang Q.-Q. Shen X. Z. Giani J. F. Zhao T. V. Shi P. (2018). Angiotensin-converting enzyme inhibitor works as a scar formation inhibitor by down-regulating Smad and TGF-β-activated kinase 1 (TAK1) pathways in mice: ACEI for scar formation. Br. J. Pharmacol. 175, 4239–4252. 10.1111/bph.14489 30153328 PMC6193878

[B135] Townend J. N. West J. N. Davies M. K. Littler W. A. (1992). Effect of quinapril on blood pressure and heart rate in congestive heart failure. Am. J. Cardiol. 69, 1587–1590. 10.1016/0002-9149(92)90708-7 1598874

[B136] Trikha R. Greig D. Kelley B. V. Mamouei Z. Sekimura T. Cevallos N. (2020). Inhibition of angiotensin converting enzyme impairs anti-staphylococcal immune function in a preclinical model of implant infection. Front. Immunol. 11, 1919. 10.3389/fimmu.2020.01919 33042111 PMC7518049

[B137] Trump S. Lukassen S. Anker M. S. Chua R. L. Liebig J. Thürmann L. (2021). Hypertension delays viral clearance and exacerbates airway hyperinflammation in patients with COVID-19. Nat. Biotechnol. 39, 705–716. 10.1038/s41587-020-00796-1 33361824

[B138] Tu M. Wang C. Chen C. Zhang R. Liu H. Lu W. (2018). Identification of a novel ACE-inhibitory peptide from casein and evaluation of the inhibitory mechanisms. Food Chem. 256, 98–104. 10.1016/j.foodchem.2018.02.107 29606478

[B139] Turner A. J. Hooper N. M. (2002). The angiotensin–converting enzyme gene family: Genomics and pharmacology. Trends Pharmacol. Sci. 23, 177–183. 10.1016/s0165-6147(00)01994-5 11931993

[B140] Tzakos A. G. Galanis A. S. Spyroulias G. A. Cordopatis P. Manessi-Zoupa E. Gerothanassis I. P. (2003). Structure-function discrimination of the N- and C- catalytic domains of human angiotensin-converting enzyme: Implications for Cl- activation and peptide hydrolysis mechanisms. Protein Eng. 16, 993–1003. 10.1093/protein/gzg122 14983080

[B141] Tzakos A. G. Gerothanassis I. P. (2005). Domain-selective ligand-binding modes and atomic level pharmacophore refinement in angiotensin I converting enzyme (ACE) inhibitors. Chembiochem. 6, 1089–1103. 10.1002/cbic.200400386 15883972

[B142] Ustaoğlu G. Erdal E. Karaş Z. (2021). Influence of different anti-hypertensive drugs on gingival overgrowth: A cross‐sectional study in a Turkish population. Oral Dis. 27, 1313–1319. 10.1111/odi.13655 32991012

[B143] van Esch J. H. M. Tom B. Dive V. Batenburg W. W. Georgiadis D. Yiotakis A. (2005). Selective angiotensin-converting enzyme C-domain inhibition is sufficient to prevent angiotensin I–induced vasoconstriction. Hypertension 45, 120–125. 10.1161/01.HYP.0000151323.93372.f5 15583077

[B144] Verdecchia P. Angeli F. Reboldi G. (2018). Hypertension and atrial fibrillation: Doubts and certainties from basic and clinical studies. Circ. Res. 122, 352–368. 10.1161/CIRCRESAHA.117.311402 29348255

[B145] Wang J. J. Edin M. L. Zeldin D. C. Li C. Wang D. W. Chen C. (2020). Good or bad: Application of RAAS inhibitors in COVID-19 patients with cardiovascular comorbidities. Pharmacol. Ther. 215, 107628. 10.1016/j.pharmthera.2020.107628 32653530 PMC7346797

[B146] Wang L. Wu W. (2019). Angiotensin-converting enzyme inhibiting ability of ethanol extracts, steviol glycosides and protein hydrolysates from stevia leaves. Food Funct. 10, 7967–7972. 10.1039/c9fo02127b 31750488

[B147] Warner F. J. Rajapaksha H. Shackel N. Herath C. B. (2020). ACE2: From protection of liver disease to propagation of COVID-19. Clin. Sci. 134, 3137–3158. 10.1042/CS20201268 33284956

[B148] Wei L. Alhenc-Gelas F. Corvol P. Clauser E. (1991). The two homologous domains of human angiotensin I-converting enzyme are both catalytically active. J. Biol. Chem. 266, 9002–9008. 10.1016/s0021-9258(18)31543-6 1851160

[B149] Wei L. Clauser E. Alhenc-Gelas F. Corvol P. (1992). The two homologous domains of human angiotensin I-converting enzyme interact differently with competitive inhibitors. J. Biol. Chem. 267, 13398–13405. 10.1016/s0021-9258(18)42224-7 1320019

[B150] Weir M. R. Rolfe M. (2010). Potassium homeostasis and renin-angiotensin-aldosterone system inhibitors. Clin. J. Am. Soc. Nephrol. 5, 531–548. 10.2215/CJN.07821109 20150448

[B151] Weller H. N. Gordon E. M. Rom M. B. Pluščec J. (1984). Design of conformationally constrained angiotensin-converting enzyme inhibitors. Biochem. Biophys. Res. Commun. 125, 82–89. 10.1016/s0006-291x(84)80337-x 6095846

[B152] Williams T. A. Michaud A. Houard X. Chauvet M.-T. Soubrier F. Corvol P. (1996). *Drosophila melanogaster* angiotensin I-converting enzyme expressed in Pichia pastoris resembles the C domain of the mammalian homologue and does not require glycosylation for secretion and enzymic activity. Biochem. J. 318, 125–131. 10.1042/bj3180125 8761461 PMC1217597

[B153] Wu J.-S. Li J.-M. Lo H.-Y. Hsiang C.-Y. Ho T.-Y. (2020). Anti-hypertensive and angiotensin-converting enzyme inhibitory effects of Radix Astragali and its bioactive peptide AM-1. J. Ethnopharmacol. 254, 112724. 10.1016/j.jep.2020.112724 32119952

[B154] Wu J. Hall A. S. Gale C. P. AIREX Study Investigators (2021a). Long-term survival benefit of ramipril in patients with acute myocardial infarction complicated by heart failure. Heart 107, 389–395. 10.1136/heartjnl-2020-316823 33452123

[B155] Wu Q. Luo F. Wang X.-L. Lin Q. Liu G.-Q. (2021b). Angiotensin I-converting enzyme inhibitory peptide: An emerging candidate for vascular dysfunction therapy. Crit. Rev. Biotechnol. 42, 736–755. 10.1080/07388551.2021.1948816 34634988

[B156] Wu Q. Luo F. Wang X.-L. Lin Q. Liu G.-Q. (2022). Angiotensin I-converting enzyme inhibitory peptide: An emerging candidate for vascular dysfunction therapy. Crit. Rev. Biotechnol. 42, 736–755. 10.1080/07388551.2021.1948816 34634988

[B157] Wu S. Feng X. Lan X. Xu Y. Liao D. (2015). Purification and identification of Angiotensin-I Converting Enzyme (ACE) inhibitory peptide from lizard fish (Saurida elongata) hydrolysate. J. Funct. Foods 13, 295–299. 10.1016/j.jff.2014.12.051

[B158] Wzgarda A. Kleszcz R. Prokop M. Regulska K. Regulski M. Paluszczak J. (2017). Unknown face of known drugs – what else can we expect from angiotensin converting enzyme inhibitors? Eur. J. Pharmacol. 797, 9–19. 10.1016/j.ejphar.2016.12.031 28087255

[B159] Yan J.-K. Ding L.-Q. Shi X.-L. Donkor P. O. Chen L.-X. Qiu F. (2017). Megastigmane glycosides from leaves of Eucommia ulmoides Oliver with ACE inhibitory activity. Fitoterapia 116, 121–125. 10.1016/j.fitote.2016.12.001 27923676

[B160] Yancy C. W. Jessup M. Bozkurt B. Butler J. Casey D. E. Colvin M. M. (2017). 2017 ACC/AHA/HFSA focused update of the 2013 ACCF/AHA guideline for the management of heart failure: A report of the American college of cardiology/American heart association task force on clinical practice guidelines and the heart failure society of America. Circulation 136, e137–e161. 10.1161/CIR.0000000000000509 28455343

[B161] Yang G. Tan Z. Zhou L. Yang M. Peng L. Liu J. (2020a). Effects of angiotensin II receptor blockers and ACE (Angiotensin-Converting enzyme) inhibitors on virus infection, inflammatory status, and clinical outcomes in patients with COVID-19 and hypertension: A single-center retrospective study. Hypertension 76, 51–58. 10.1161/HYPERTENSIONAHA.120.15143 32348166

[B162] Yang J. Yang X. Gao L. Zhang J. Yi C. Huang Y. (2021). The role of the renin-angiotensin system inhibitors in malignancy: A review. Am. J. Cancer Res. 11, 884–897. 33791161 PMC7994166

[B163] Yang Y. Ma L. Xu Y. Liu Y. Li W. Cai J. (2020b). Enalapril overcomes chemoresistance and potentiates antitumor efficacy of 5-FU in colorectal cancer by suppressing proliferation, angiogenesis, and NF-κB/STAT3-regulated proteins. Cell Death Dis. 11, 477. 10.1038/s41419-020-2675-x 32581212 PMC7314775

[B164] Yao J. Gong X. Shi X. Fan S. Chen J. Chen Q. (2020). The efficacy of angiotensin converting enzyme inhibitors versus angiotensin II receptor blockers on insulin resistance in hypertensive patients: A protocol for a systematic review and meta-analysis. Medicine 99, e20674. 10.1097/MD.0000000000020674 32541513 PMC7302663

[B165] Zhang P. Zhu L. Cai J. Lei F. Qin J.-J. Xie J. (2020a). Association of inpatient use of angiotensin-converting enzyme inhibitors and angiotensin II receptor blockers with mortality among patients with hypertension hospitalized with COVID-19. Circ. Res. 126, 1671–1681. 10.1161/CIRCRESAHA.120.317134 32302265 PMC7265882

[B166] Zhang X. Yu J. Pan L.-Y. Jiang H.-Y. (2020b). ACEI/ARB use and risk of infection or severity or mortality of COVID-19: A systematic review and meta-analysis. Pharmacol. Res. 158, 104927. 10.1016/j.phrs.2020.104927 32422341 PMC7227582

[B167] Zhang Y. Cong J. Bao G. Gu S. Wang X. (2022). *In vitro* gastrointestinal digestion study and identification of novel angiotensin i-converting enzyme inhibitory peptide from broccoli (brassica oleracea). LWT 164, 113613. 10.1016/j.lwt.2022.113613

[B168] Zhang Y. Ding X. Hua B. Liu Q. Chen H. Zhao X.-Q. (2020c). Real-world use of ACEI/ARB in diabetic hypertensive patients before the initial diagnosis of obstructive coronary artery disease: Patient characteristics and long-term follow-up outcome. J. Transl. Med. 18, 150. 10.1186/s12967-020-02314-y 32238168 PMC7114815

[B169] Zhao Y. Xu C. (2008). Structure and function of angiotensin converting enzyme and its inhibitors. Chin. J. Biotechnol. 24, 171–176. 10.1016/s1872-2075(08)60007-2 PMC7148949 18464595

